# Dysregulation of RNA splicing in early non-alcoholic fatty liver disease through hepatocellular carcinoma

**DOI:** 10.1038/s41598-024-52237-7

**Published:** 2024-01-30

**Authors:** Nicholas J. G. Webster, Deepak Kumar, Panyisha Wu

**Affiliations:** 1 Jennifer Moreno VA Medical Center, San Diego, CA 92161 USA; 2 Division of Endocrinology and Metabolism, Department of Medicine, University of California, San Diego, CA 92093 USA; 3 Moores Cancer Center, University of California, San Diego, CA 92093 USA

**Keywords:** Liver cancer, Hepatocellular carcinoma, Non-alcoholic fatty liver disease, Non-alcoholic steatohepatitis, Alternative splicing, RNA splicing

## Abstract

While changes in RNA splicing have been extensively studied in hepatocellular carcinoma (HCC), no studies have systematically investigated changes in RNA splicing during earlier liver disease. Mouse studies have shown that disruption of RNA splicing can trigger liver disease and we have shown that the splicing factor SRSF3 is decreased in the diseased human liver, so we profiled RNA splicing in liver samples from twenty-nine individuals with no-history of liver disease or varying degrees of non-alcoholic fatty liver disease (NAFLD). We compared our results with three publicly available transcriptome datasets that we re-analyzed for splicing events (SEs). We found many changes in SEs occurred during early liver disease, with fewer events occurring with the onset of inflammation and fibrosis. Many of these early SEs were enriched for SRSF3-dependent events and were associated with SRSF3 binding sites. Mapping the early and late changes to gene ontologies and pathways showed that the genes harboring these early SEs were involved in normal liver metabolism, whereas those harboring late SEs were involved in inflammation, fibrosis and proliferation. We compared the SEs with HCC data from the TCGA and observed that many of these early disease SEs are found in HCC samples and, furthermore, are correlated with disease survival. Changes in splicing factor expression are also observed, which may be associated with distinct subsets of the SEs. The maintenance of these SEs through the multi-year oncogenic process suggests that they may be causative. Understanding the role of these splice variants in metabolic liver disease progression may shed light on the triggers of liver disease progression and the pathogenesis of HCC.

## Introduction

Alternative RNA splicing is a crucial post-transcriptional process that plays a fundamental role in the regulation of gene expression and protein diversity. It allows a single gene to generate multiple mRNA transcripts by selectively including or excluding exons, or portions of exons, during processing of the primary RNA transcript. Alternative splicing has emerged as a key mechanism influencing the development and progression of liver diseases^1^. Understanding the intricate interplay between alternative splicing and liver pathology is essential for uncovering novel therapeutic targets and advancing personalized medicine approaches for these conditions^2^. Liver disease encompasses a wide range of conditions, such as viral hepatitis, non-alcoholic fatty liver disease (NAFLD), alcoholic liver disease (ALD), and cirrhosis^3–6^. Alternative splicing has been implicated in the regulation of critical genes and pathways associated with liver diseases^7–10^. For instance, aberrant splicing events in genes involved in hepatic lipid metabolism can contribute to the pathogenesis of NAFLD and ALD^11^. Additionally, dysregulation of splicing factors, which control the splicing process, has been observed in liver diseases, indicating their potential as diagnostic markers or therapeutic targets^12^.

Liver cancer, predominantly hepatocellular carcinoma (HCC), is a major global health concern with limited treatment options and poor prognosis. Alternative splicing also plays a significant role in the development and progression of HCC by generating transcript isoforms that contribute to tumor heterogeneity and altered cellular functions^13–18^. Abnormal splicing events can affect genes involved in cell cycle regulation, apoptosis, cell adhesion, and angiogenesis, all of which are crucial processes for tumor growth and metastasis^19^. Furthermore, alternative splicing can lead to the production of tumor-specific antigens or fusion proteins that have potential diagnostic or therapeutic implications. Targeting splicing events associated with liver cancer holds promise for developing novel precision medicine approaches and improving patient outcomes.

Recent advancements in high-throughput sequencing technologies and bioinformatics tools have facilitated comprehensive analyses of transcriptional changes in liver disease and liver cancer. Moreover, the integration of transcriptomic, proteomic, and genomic data has provided valuable insights into the pathogenesis of liver disease^20–22^. Most of these studies, however, have focused on gene or protein level expression and have ignored transcriptional or protein isoforms and their complexities^23–26^. Recent studies in mice have shown that RNA splicing is crucial for liver maturation and function but studies in humans are lacking^27–29^. These studies have revealed complex splicing patterns, unique splice variants, and splicing factor dysregulation specific to the liver^11,30–33^. However, further investigation is necessary to decipher the regulatory mechanisms underlying alternative splicing in liver diseases in humans and identify potential therapeutic targets. A deeper understanding of the role of alternative splicing in liver disease and cancer will pave the way for the development of precision medicine strategies tailored to individual patients. To this end, we profiled alternative splicing during early liver disease in patients with non-viral, non-alcoholic fatty liver disease (NAFLD) and non-alcoholic steatohepatitis (NASH).

## Results

### Changes in alternative splicing are enriched for SRSF3-dependent splicing events

**Figure 1:**
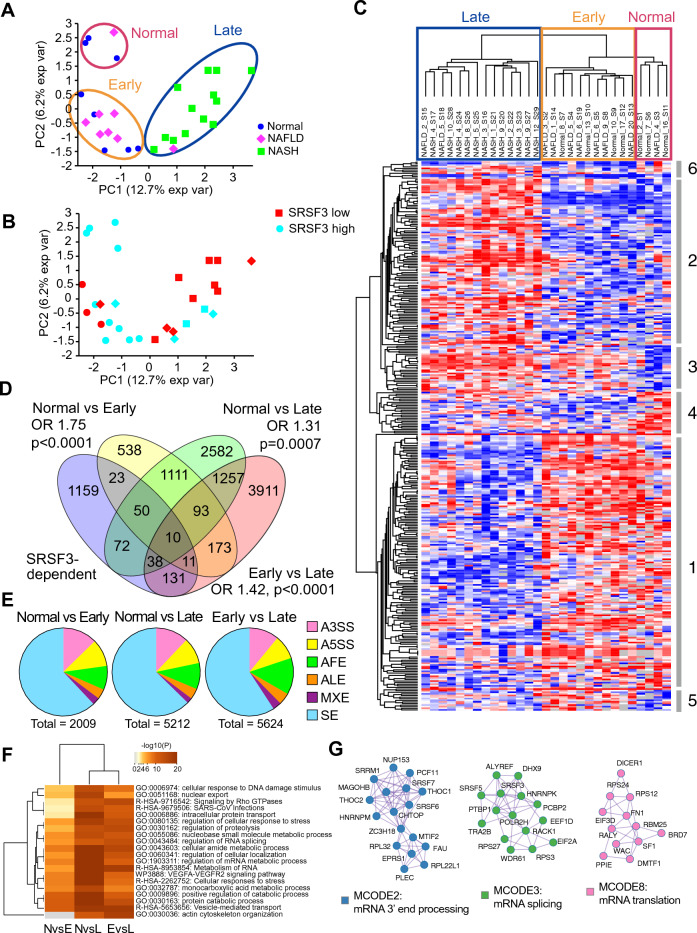
RNA splicing is altered in NAFLD and NASH: (**A**) PCA plot based on the analysis of alternative splicing events in RNA from liver samples from 8 individuals with normal liver (blue), 8 individuals with NAFLD (magenta) and 13 individuals with NASH (green). The percent-spliced-in (PSI) value for each event was calculated with Psichomics. K-means clustering highlighted three distinct clusters that are circled, which we have labelled Normal, Early or Late. (**B**) PCA plot color-coded for SRSF3 protein expression by western blot. SRSF3 expression below the mean is colored red, high expression above the mean is labeled cyan. (**C**) Heatmap of the top 500 SEs with < 5 missing values and PSI variance > 0.01. The samples clustered into the same three groups as the PCA. Red indicates high PSI and blue low PSI. (**D**) Venn diagram showing overlap of the statistically different SEs from the pairwise clusters comparisons and the set of SRSF3-dependent SEs from shRNA knockdown of SRSF3 in HepG2 cells. Enrichment for SRSF3-dependent SEs is indicated by the odds ratio (OR, χ^2^ p value). (**E**) Pie charts showing distribution of splicing event types between clusters: alternative 3′ splice site (A3SS), alternative 5′-splice site (A5SS), alternative first exon (AFE), alternative last exon (ALE), mutually-exclusive exons (MXE) and skipped exons (SE). (**F**) Heatmap showing enriched GO ontology terms from genes harboring the SEs. Color coding shows -log10 p-value for enrichment. NvsE, Normal vs Early; NvsL Normal vs Late; EvsL Early vs Late cluster. (**G**) Protein–protein MCODE interaction networks based on spliced genes. Nodes are shown as circles, interactions as lines.

We have previously reported that the splicing factor Ser/Arg-rich splicing factor 3 (SRSF3) is proteolytically degraded in fatty liver disease^29^, so we obtained 43 frozen liver samples of individuals with no known history of liver disease, or histologically verified non-alcoholic fatty liver disease (NAFLD) or non-alcoholic steatohepatitis (NASH) from the University of Minnesota Liver Tissue and Cell Distribution Service. We were able to extract high-quality RNA from 29 of these samples (13 no history, 7 NAFLD, 9 NASH) which was then subjected to high-throughput short-read sequencing. The histology of the samples was confirmed by a pathologist or assessed for those samples without pathology information (Table S1). This resulted in the identification of 8 samples with normal liver histology, 8 samples with NAFLD and 13 samples with NASH. Reads were aligned to the human hg38 genome with STAR and gene count and splice junction files analyzed with Psichomics^34^. Principal component analysis (PCA) of the splicing data (percent-spliced-in or PSI index) indicated the presence of three clusters of samples. One cluster contained three normal samples and one NAFLD, the second was a mixture of 5 normal and 6 NAFLD samples, and the third 1 NAFLD and 13 NASH (Fig. 1A). The clusters were labeled Normal, Early and Late disease, respectively, for further analysis. The contribution of PC1 and PC2 were modest (only 12.5% and 6.2%) on a dataset that contained 78,156 alternative splicing events (SEs) derived from 753,516 splice junctions, which was likely due to the sparsity of values for many SEs. So, we used dimension reduction to filter out SEs with more than 5 missing values reducing the dataset to 6797 SEs. When these were used for PCA analysis the relative contribution of PC1 increased to 29.2% indicating that the major difference was related to the progression from normal/early disease to late disease (Fig. S1A). We also plotted the PCA data by SRSF3 expression level, which we had previously measured by western blot^29^ (Fig. 1B). The Normal cluster showed high SRSF3 expression, whereas the Late cluster showed predominantly low SRSF3 expression, with the Early cluster showing mixed expression. The SRSF3 protein level was significantly lower in the Late cluster than the Normal cluster (Fig. S1B). We assessed which SEs contributed to the PCA distribution (Table S2) and quantification of the top two contributing SEs indicated the use of an alternative first exon in the Late cluster in the TPM1 and SLCO2B1 transcripts (Fig. S1C). To visualize the variation in splicing across the samples we used unsupervised clustering of the top 500 SEs with < 5 missing values and a PSI variance > 0.01 (Fig. 1C). The samples clustered the same way as the PCA analysis with the Late cluster showing greatest dissimilarity to the Normal and Early clusters. The SEs clustered into six groups: two large groups 1 and 2 were SEs that were different in the Late cluster representing changes that occur in later disease; two groups 3 and 4 were SEs different in the Normal cluster representing changes that occur in early disease (Fig. S1D); and two small groups 5 and 6 which contained SEs that were only different in the Early cluster representing SEs that changed early in disease but were not maintained through later disease (Fig. S1E). To identify which SEs were significantly altered we performed a statistical comparison of all three clusters using the Kruskal–Wallis rank sum test (Table S3, 4652 significant SEs), and then compared individual clusters using t-test and Wilcoxon rank sum tests (Tables S4–6). We also analyzed a dataset from the ENCODE consortium, in which SRSF3 had been knocked down in human HepG2 hepatoma cells, to provide a list of SRSF3-dependent events (Table S7)^35^. The SEs were filtered to only analyze events that were detected in both human liver and HepG2 cells (2872 sig SEs, 37,426 total SEs). SEs that were significantly different between pairs of clusters (p < 0.05) were then overlapped with each other and with SRSF3-dependent events from HepG2 cells (Fig. 1D). SRSF3-dependent SEs were significantly enriched in the overall cluster data (2872 SE, odds ratio OR 1.54, p < 0.0001 by χ^2^) and were also enriched in the individual cluster comparisons, with enrichment being greatest for the Early cluster compared to the Normal cluster (OR 1.75, p < 0.0001). Changes in exon skipping were the predominant form of alternative splicing in all three comparisons followed by alternative 3′ splice sites and first exons (Fig. 1E).

To gain an understanding of the functional effect of these changes we mapped the genes affected by the SEs to pathways and gene ontologies using Metascape^36^. The genes affected by these SE changes also showed considerable overlap between clusters (Fig. S1F). Nineteen of the top 20 enriched terms were shared between clusters with RNA metabolism, response to stress, protein catabolism and transport being the most significant (Fig. 1F). We also created protein–protein interaction networks out of these genes using the Molecular Complex Detection algorithm (MCODE) and the STRING and BioGRID databases^37–39^. Three of the top 8 MCODE interaction networks were involved in mRNA splicing, 3′ end processing and translation (Fig. 1G). Other networks involved organelle fusion and vesicle transport, carbohydrate metabolism, MAPK signaling and cell cycle (Fig. S1G). Inspection of the networks involved in RNA processing highlighted several splicing factors that were themselves subject to alternative splicing including SRSF3, SRSF6 and SRSF7, which contain poison exons causing non-sense mediated decay of the transcript, the spliceosome component SNRNP70, the ribosome subunit RPS24, and SRRM1 that bridges SR proteins to the snRNP complex (Fig. S1H).

**Figure 2:**
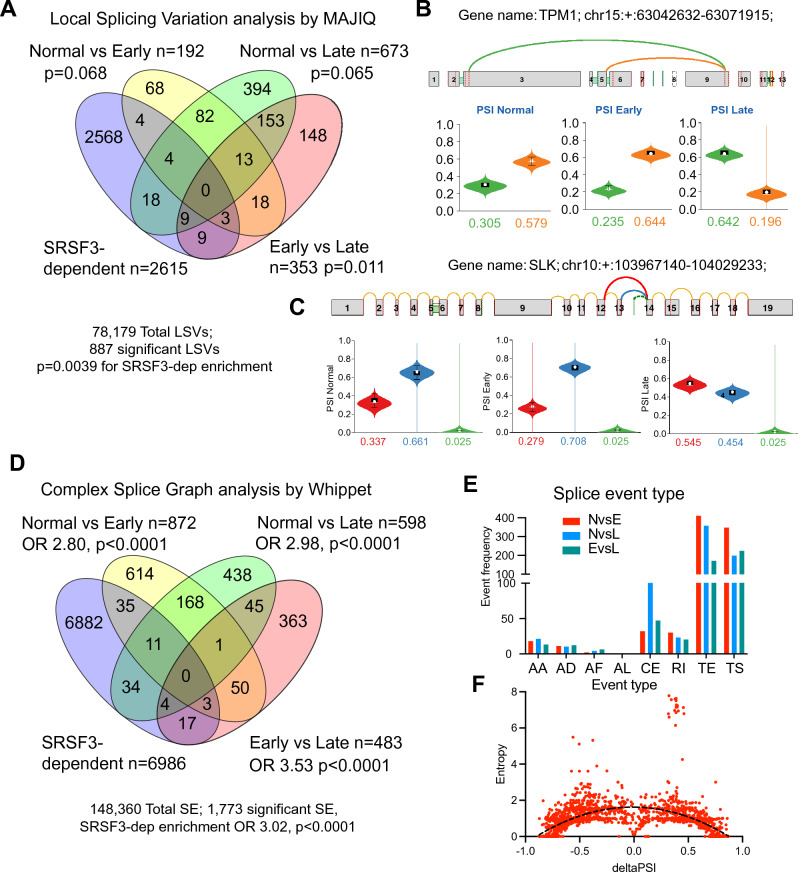
Changes in RNA splicing by splice graph. (**A**) Venn diagram showing overlap of local splicing variants (LSV) by MAJIQ (78,179 total LSVs, 887 significant LSVs). Overlap and enrichment χ^2^ p-value with LSVs from SRSF3 shRNA knockdown in HepG2 cells is shown for individual cluster comparisons (Normal, Early or Late) and overall. (**B**) Change in upstream exon utilization in *TPM1* RNA. Use of upstream exon splice site is shown in green, downstream exon in orange. Graphs underneath show PSI values for each event in the three clusters. (**C**) Skipped exon 13 in the *SLK* RNA. Exon skipping is shown in red, inclusion in blue. Green indicates a cryptic splice exon. (**D**) Venn diagram showing overlap of alternative splicing by Whippet (148,360 total SEs, 1773 significant SEs). Overlap and enrichment χ^2^ p-value with SEs from SRSF3 shRNA knockdown in HepG2 cells is shown for individual cluster comparisons and overall. (**E**) Distribution of significant splicing events across cluster comparisons. AA alternative splice acceptor, AD alternative splice donor, AF alternative first exon, AL alternative last exon, CE cassette exon, RI retained intron, TE transcriptional termination exon, and TS transcriptional start site. NvsE, Normal vs Early; NvsL Normal vs Late; EvsL Early vs Late cluster. (**F**) Graph of splice event entropy (complexity) versus PSI for significant SEs. Dotted line is quadratic curve fit (p < 0.0001).

The Psichomics algorithm analyzes individual splicing events but does not quantify more complex splicing variation. To assess more complex splicing patterns, we took two approaches based on the generation of complex splice graphs. These approaches use graph theory to derive alternative routes to generate known and novel splicing isoforms. Analysis of the clusters using MAJIQ^40^ highlighted a smaller number of local splicing variations (LSVs) that were derived from the mapping of multiple splice junctions to the splice graphs (Tables S8–10). Out of 78,179 LSVs, 887 were significantly altered between the clusters and they were significantly enriched for SRSF3-dependent events as before (OR 1.58, p = 0.0039) (Fig. 2A). Comparison of the Early and Late clusters showed significant enrichment (p = 0.011) of SRSF3-dependent splicing events, but the other comparisons did not (p = 0.068 and 0.065). As an example, the TPM1 splicing event that contributed most to the PCA analysis was also found in the LSVs showing a switch in splicing of upstream exons in the Late cluster using a more proximal first exon (Fig. 2B). Gratifyingly, known SRSF3-dependent events were also found. Splicing of exon 13 in the SLK1 gene was altered with greater skipping of this exon in the Late cluster (Fig. 2C). The genes affected by these LSVs also showed overlap with the greatest number of changes occurring between the Normal and Late clusters (Fig. S2A). The functional effect of the genes harboring these splicing events was again assessed using Metascape. Early changes were enriched for genes in metabolic pathways and networks including blood clotting, nitrogen and organic acid metabolism, hormone stimulation and cell shape (Fig. S2B and C) whereas later changes between the Normal or Early clusters and the Late cluster showed enrichment for genes in RNA processing including splicing, miRNA production, ribonucleoprotein complex assembly, and nucleocytoplasmic transport, and genes involved in regulating cell shape including morphogenesis, actin cytoskeletal rearrangement and cell-substrate interactions. Protein–protein interaction network analysis highlighted networks involved in mRNA splicing, cytoskeletal rearrangement, vesicle trafficking, mitotic centrosomes and xenobiotic metabolism (Fig. S2D).

A similar splice graph analysis was performed using Whippet^41^ and comparison with the ENCODE data again showed enrichment for SRSF3-dependent events in the overall dataset (Fig. 2D, Tables S11–13). Interestingly, this approach found more differences between the Normal and Early clusters (Figs. 2D and S2E). This analysis identifies alternative transcription start and termination sites in addition to the other SEs, and these events were more frequent than cassette exon skipping that was the major class in the Psichomics analysis (Fig. 2E). The analysis also provides a measure of splicing entropy that is related to the complexity of the splicing event. Interestingly, the splicing events that showed the greatest change in PSI had lower entropy, i.e. simpler splicing events tended to show greater changes (Fig. 2F). Functional analysis highlighted terms and pathways involving lipid metabolism, phospholipase signaling, and small molecule hydroxy acid metabolism in the early changes, with enrichment of immune response, vesicle transport, complement, chromatin modification, and mitosis in later changes (Fig. S2F and G). Protein–protein interaction network mapping with the genes harboring high entropy splicing events highlighted interaction networks incorporating proteins with known functions in the liver. The largest network included proteins involved in meta-phase progression and was centered on beta-catenin, which is the most frequently mutated gene in HCC (Fig. S2H). Other networks involved PI3K-Akt signaling, mRNA splicing and translation. These analyses showed that changes in early disease samples tended to be in metabolic pathways suggesting that changes in liver metabolism are some of the early steps, whereas changes in later disease reflected cytoskeletal and matrix changes as well as immune responses. Some of these may reflect changes in the hepatocytes but could also be indicative of the increased immune cell infiltration, stellate cell activation and fibrosis that occurs in later disease. We were not able to compare the three sets of predicted splicing events directly as each algorithm uses a different naming system and chromosomal coordinates. We could, however, compare the genes containing the alternatively spliced events; Psichomics detected the greatest number of alternatively spliced genes (2912), which included 48% of the genes detected by Majiq but only 20% of the genes detected by Whippet.

### Changes in gene expression are not enriched for SRSF3-dependent events

**Figure 3:**
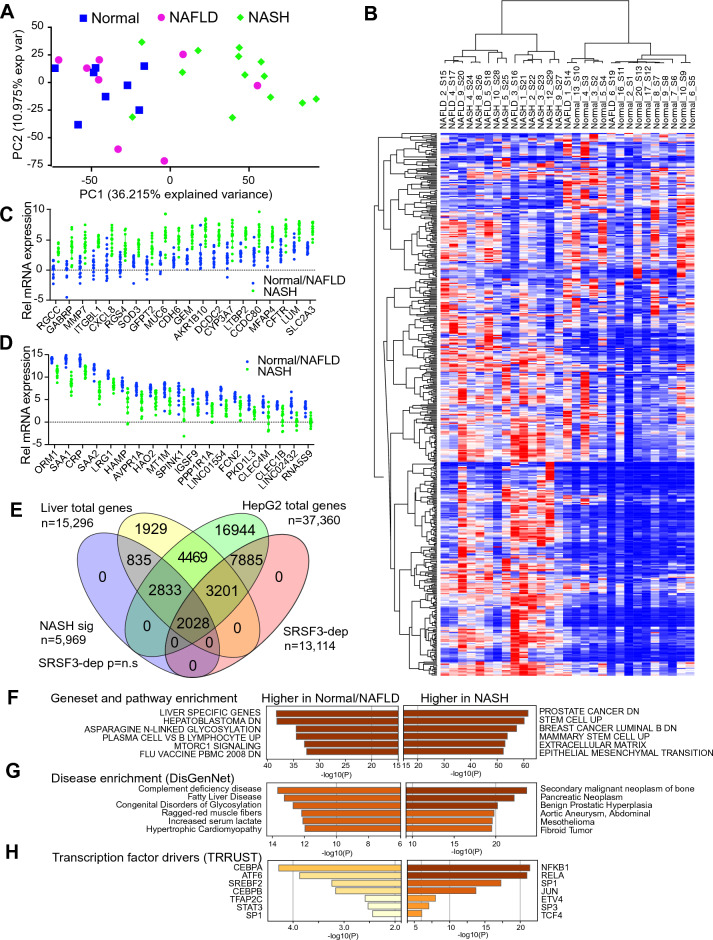
Transcriptional changes in liver disease. (**A**) PCA plot based on gene expression levels from gene-wise linear model fit and moderated t-test by empirical Bayes. Individuals with no history of liver disease (blue), individuals with NAFLD (magenta) and individuals with NASH (green). (**B**) Heatmap from unsupervised clustering of gene expression data. Red indicates high and blue low expression. Samples (**C**) Relative mRNA expression of the top twenty genes showing increased expression in the NASH cluster (green) versus the Normal/NAFLD cluster (blue). (**D**) Relative mRNA expression of the top twenty genes showing decreased expression in the NASH cluster (green) versus the normal/NAFLD cluster (blue). (**E**) Venn diagram showing overlap of gene expression showing total and significant liver genes (NASH vs Normal/NAFLD) and total and significant gene from SRSF3 shRNA knockdown in HepG2 cells. -log10 P value is shown on x axis, color indicates level of significance. (**F**) Geneset and pathway enrichment for gene higher in Normal/NAFLD vs higher in NASH. (**G**) Disease enrichment using DisGenNet for genes higher in NAFLD/Normal vs higher in NASH. (**H**) Transcription factors driving altered gene expression in Normal/NAFLD vs NASH from TRRUST.

We also analyzed changes in gene expression in these samples (Table S14). PCA analysis did not reveal the same separation as was observed with the splicing events (Fig. 3A) nor the same dependence on SRSF3 protein levels (Fig. S3A). Hierarchical clustering of the expression data, however, did show a separation of the NASH samples versus the Normal/NAFLD samples (Fig. 3B). The genes that contributed most to the PCA were distinct from the splicing events with many genes involved in inflammation and the immune response (Fig. S3C, Table S15). We also used t-distributed stochastic neighbor embedding (tSNE) (Fig. S3D) based on the expression levels and this confirmed that the samples split into two groups, the NASH group equivalent to the Late splicing cluster, and a normal/NAFLD group equivalent to the Normal and Early splicing clusters (Fig. S3B). Interestingly, six of the 20 most significant up-regulated genes in NASH encoded proteins involved in the extracellular matrix (*MMP7, ITGBL1, LUM, CCDC80, CDH6, MUC6*) consistent with fibrosis (Fig. 3C), whereas eight of the twenty top down-regulated genes (*ORM1, SAA1, CRP, SAA2, LRG1, HAMP, SPINK1, FCN2*) encode for secreted proteins suggesting impaired hepatocyte secretory function (Fig. 3D). We analyzed the changes in gene expression between the Normal/NAFLD group and the NASH group then compared those to the SRSF3-dependent gene expression changes from the ENCODE dataset in HepG2 cells (Table S16). The transcriptomes of the liver samples and the HepG2 cells only showed partial overlap with 82% of liver genes being detected in HepG2 cells but only 34% of HepG2 genes being detected in primary liver samples (Fig. 3E). Unlike the splicing events, SRSF3-dependent gene expression changes were not significantly enriched in the NASH-dependent genes (42% SRSF3-dep, p = 0.98 by χ^2^ test). We subjected the top 1000 genes that were over-expressed or under-expressed in NASH to pathway and network mapping. The genes that were higher in the Normal/NAFLD group were enriched for genesets and pathways involved in liver function or were decreased in hepatoblastoma, whereas the genes increased in NASH were enriched for cancer, stem cell, and extracellular matrix pathways (Fig. 3F). Mapping the differential expression to the DisGenNet disease signature database^42^ showed that the Normal/NAFLD genes were enriched for signatures of complement deficiency and fatty liver disease, whereas the NASH genes were enriched for neoplastic signatures (Fig. 3G) and analysis of the altered genes for transcription factor drivers showed that genes in the Normal/NAFLD group were driven by metabolism related transcription factors such as C/EBPα/β, ATF6 and SREBP2, whereas genes in the NASH group were driven by inflammatory pathways such as NFκB, and RelA (Fig. 3H).

**Figure 4:**
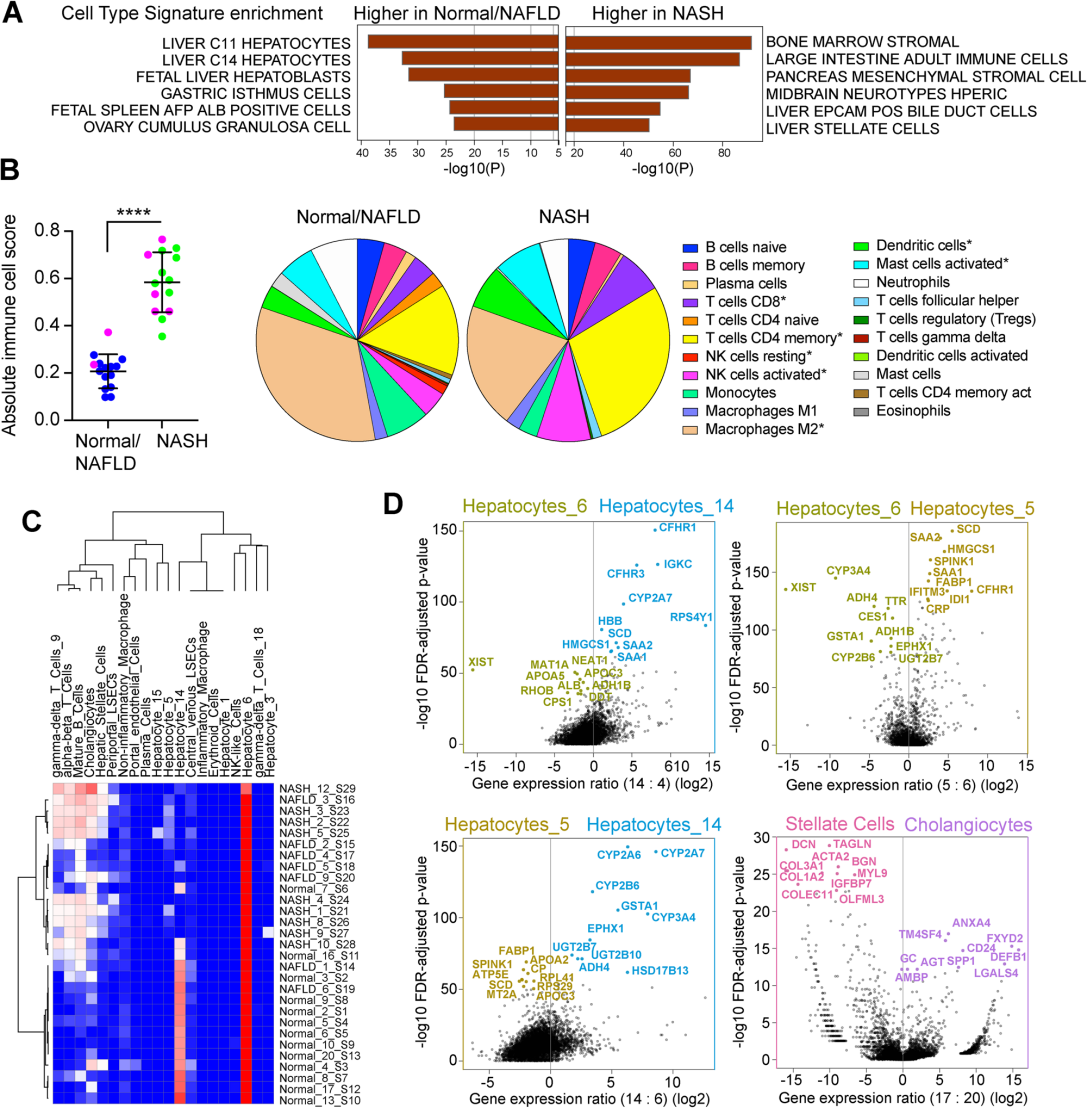
Liver and immune cells signatures. (**A**) Enrichment of cell-type signatures in gene higher in Normal/NAFLD vs higher in NASH. -log10 P value is shown on x axis. (**B**) Immune cell score and immune cell composition in Normal/NAFLD and NASH groups using CIBERSORT. Pie charts show relative immune cell composition. Asterisks indicate statistical significance difference in NASH vs Normal/NAFLD by t-test, ****p < 0.0001, *p < 0.05. (**C**) Heatmap showing unsupervised clustering of liver cell composition of each sample using CIBERSORT and the cell signatures from the Bader Lab Liver Atlas^43^. Samples cluster into Normal/NAFLD and NASH groups as before. Blue indicates low relative cell content, red high. (**D**) Pairwise comparisons of gene expression in hepatocyte clusters 6, 5 and 14 or a comparison of stellate cells vs cholangiocytes. Graphs plot -log10 FDR-adjusted P value against log2 gene expression ratio. Top differentially expressed genes are shown.

At the cell level, the genes that were higher in Normal/NAFLD were enriched for hepatocyte or hepatoblast cell signatures whereas those higher in NASH were enriched for stromal, immune, and stellate cell signatures (Fig. 4A). To confirm the identity of the clusters, the expression data was deconvoluted using CIBERSORT to predict immune cell composition^44^. The total predicted immune cell count was greater in the NASH cluster than the Normal/NAFLD, with an increase in CD8 and CD4 T cells and dendritic cells, and a decrease in M2 macrophages and mast cells confirming the hepatitis that is characteristic of NASH (Fig. 4B). The immune cell changes were maintained if the data were analyzed according to the splicing clusters (Fig. S3E). We also deconvoluted the expression data using a signature matrix generated from a single cell sequencing study of human liver (Fig. 4C)^43^. All livers showed predominance of hepatocytes (Hepatocytes_6) that are characterized by expression of *ALB, ALDH1L1, ADH1B, XIST* and *APOC3* (Figs. 4D and S3F). The Normal/NAFLD cluster samples also showed the presence of hepatocytes that have high expression of *CYP2A6, CYP2A7, CYP2B6, CFHR1/3* and *GSTA1* (Hepatocytes_14) and are characteristic of hepatocytes involved in xenobiotic, steroid, fatty acid and drug metabolism (Figs. 4D and S3F). This cluster of hepatocytes decreases in the NASH samples and is replaced by a population of hepatocytes with high expression of *HMGCS1, SCD* and *FABP1* (Hepatocytes_5) characteristic of Zone 1 hepatocytes (Figs. 4D and S3F). Similar cell populations were identified when a cell signature matrix was derived from the human normal Liver Cell Atlas sequencing study (Fig. S3G)^45^. These results indicate that the samples from NASH patients have higher immune cell infiltration, greater fibrosis, impaired secretory function, and higher neoplastic gene expression. Gene expression in the Normal/NAFLD samples is driven by liver-specific transcription factors but NASH samples are driven by inflammatory signaling transcription factors.

### Alternative splicing events correlate with SRSF3 binding

**Figure 5:**
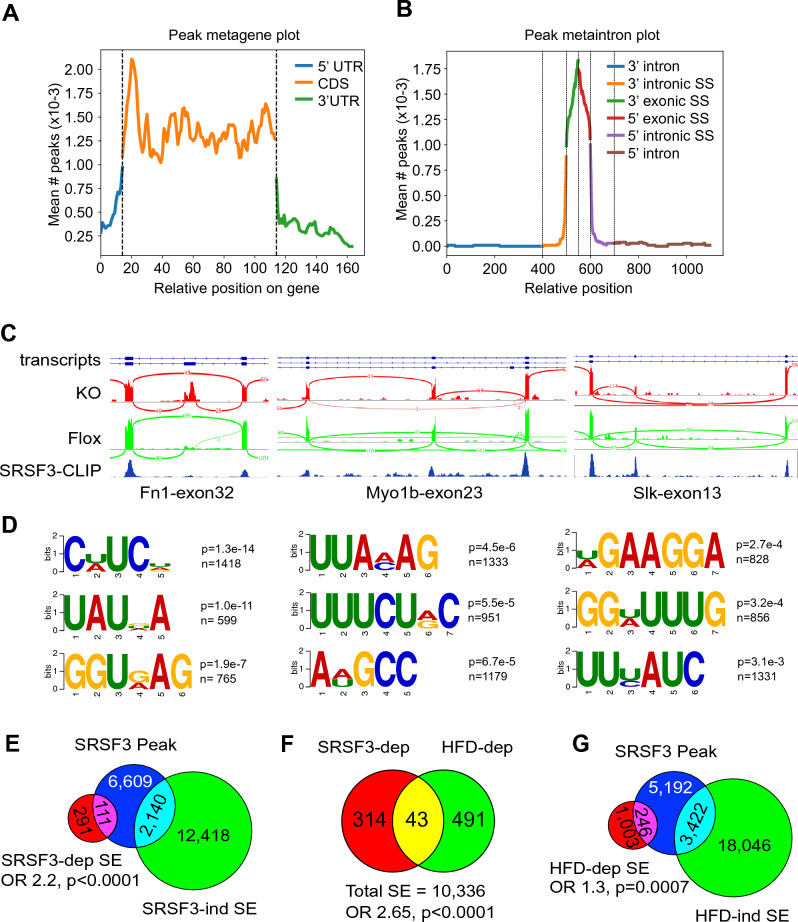
eCLIP analysis of SRSF3 binding sites in mouse hepatocytes. (**A**) Metagene plot showing distribution of SRSF3 binding peaks by eCLIP relative to gene 5-UTR, coding region or 3′UTR. (**B**) Metaintron plot showing position of SRSF3 binding peaks relative to exon/intron boundaries. (**C**) SRSF3 binding peaks in the Fn1, Myo1b and Slk compared to expression in flox and SRSF3 knockout hepatocytes. Transcript structures are shown above, sashimi plot of RNAseq reads are shown in red for SRSF3 KO and green for Flox mice, SRSF3-CLIP peaks are shown underneath in blue. (**D**) SRSF3 binding motifs from Pureclip analysis of the cross-linking sites. Enrichment p value and number of sites is given. (**E**) Overlap of SRSF3 CLIP peaks with SRSF3-dependent (Kruskal p < 0.05) and independent SEs. (**F**) Overlap of SRSF3-dependent and HFD-dependent liver splicing events. (**G**) Overlap of SRSF3 CLIP peaks with HFD-dependent (Kruskal p < 0.05) and HFD-independent SEs in liver.

As SRSF3-dependent SEs were enriched in the changes in RNA splicing during early liver disease, we sought to understand how SRSF3 could contribute to the changes in RNA splicing. We performed eCLIP with antibodies to SRSF3 in mouse primary hepatocytes^46^. Most of the binding peaks mapped to the coding-region of genes (Fig. 5A) and within 100 nucleotides of the intron splice sites (Fig. 5B). We assessed SRSF3 binding to three known target genes *Fn1, Slk* and *Myo1b* (Fig. 5C): SRSF3 represses inclusion of exon 32 in the Fn1 gene and SRSF3 showed binding to the flanking exons 31 and 33 but not to exon 32; SRSF3 also represses inclusion of exon 23 in the Myo1b gene and SRSF3 shows a strong binding peak to the upstream exon 22; in contrast SRSF3 stimulates incorporation of exon 13 in the Slk1 gene and SRSF3 showed binding to all three exons 12, 13 and 14. Analysis of the SRSF3 eCLIP data using Pureclip^47^ identified 13,119 crosslink sites encompassing 8,859 binding regions (Table S17). Sequences enriched at the crosslink sites were analyzed using DREME^48^ to identify conserved motifs and FIMO^49^ to find individual occurrences (Table S18). The top scoring motif CU/AUC was found in 1418 binding sites (p = 1.3e−14). Other motifs found include additional pyrimidine-rich motifs (UUUCUA/GC, UUU/CAUC, GGU/AUUUG) and purine-rich motifs (GAAGGA, GGUG/AAG) (Fig. 5D). The pyrimidine-rich motifs match the SRSF3 consensus motif found by SELEX (WCWWC), whereas the purine rich motifs are similar to motifs bound by other SR proteins such as SRSF1. To investigate whether SRSF3 binding correlated with SRSF3-dependent splicing we analyzed RNAseq data from mouse *Srsf3-flox* hepatocytes with Adenoviral-CRE mediated deletion of SRSF3. The RNAseq data were analyzed using the same approach used for the human samples. Splicing events were called as SRSF3-dependent if altered in the SRSF3 knockout hepatocytes with p < 0.05, and an SRSF3 binding peak had to map to < 1000 nucleotides from a splicing event for it to be called positive. SRSF3-dependent splicing events were significantly enriched for SRSF3 binding peaks (OR 2.2, p < 0.0001, Fig. 5E). We had previously published that SRSF3 protein is reduced in livers of obese mice on high-fat diet (HFD) and we recently showed that multiple lipid components of the diet can lead to proteosomal degradation of SRSF3 (Fig. S4). Consequently, we found that SRSF3-dependent splicing events were enriched in livers from the HFD mice (OR 2.65, p < 0.0001, Fig. 5F). SRSF3 peaks were also enriched in the altered splicing events in the HFD livers (OR 1.3, p = 0.0007, Fig. 5G). These results indicate that SRSF3 binding is enriched near both SRSF3- and HFD-dependent splicing events.

### Changes in alternative splicing with degree of liver disease

**Figure 6:**
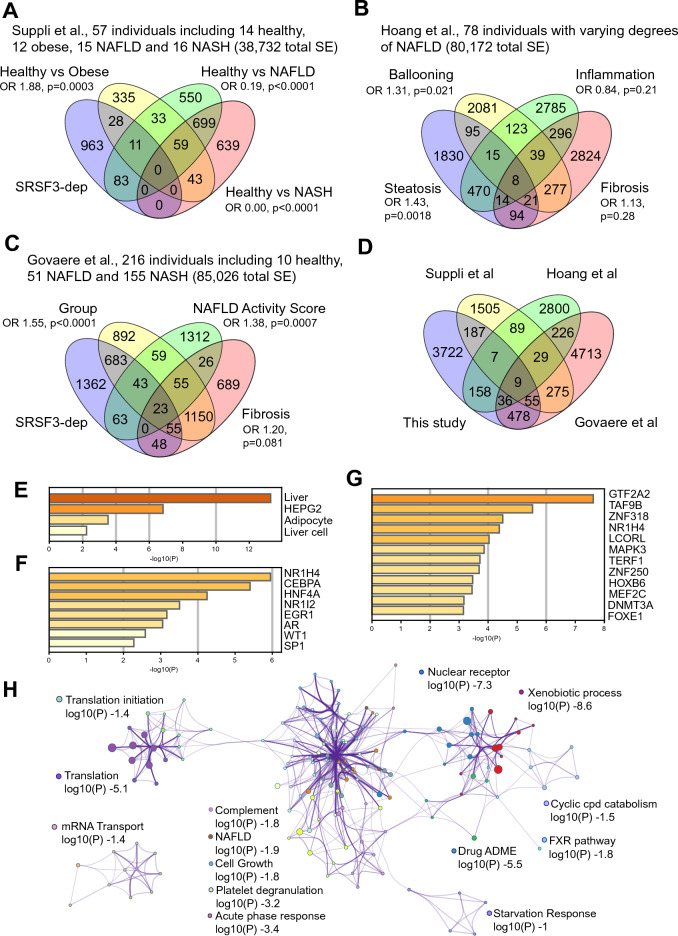
Confirmation of altered RNA splicing and SRSF3-dependence in three other NASH datasets. (**A**) Analysis of RNA splicing in Suppli et al. in 57 individuals using the PSI approach in psichomics. Venn diagram shows overlap of SEs altered in Obese, NAFLD or NASH groups relative to Healthy lean controls with SRSF3-dependent SEs from HepG2 cells. Odds ratio (OR) for enrichment of SRSF3-dep SEs in each group is shown with p value. (**B**) Analysis of RNA splicing in Hoang et al. in 78 individuals with varying degrees of NAFLD using the PSI approach. Venn diagram shows overlap of SEs altered based on steatosis score, hepatocyte ballooning, inflammation score, or fibrosis score with SRSF3-dependent SEs from HepG2 cells. (**C**) Analysis of RNA splicing in Govaere et al. of 216 individuals with NAFLD or various degrees of NASH using the PSI approach. Venn diagram shows overlap of SEs altered based on group, fibrosis score or NAFLD Activity Score with SRSF3-dependent SEs from HepG2 cells. (**D**) Overlap of altered SEs in the four studies based on a Kruskal p < 0.05 threshold. (**E**) Analysis of 136 SEs that are common to 3 or more studies by cell type. (**F**) Analysis of transcription factor driving expression of the genes harboring the 136 SEs. (**G**) Analysis of transcription factor targets of the genes with the 136 SEs. (**H**) Network showing clusters of GO terms that are significantly enriched in the 136 common SEs.

Our data suggested that changes in SRSF3-dependent splicing events could be observed in early liver disease, so we investigated alterations in RNA splicing in three other liver transcriptome datasets to see if SRSF3-dependence correlated with early versus late disease. Suppli et al. investigated the transcriptional profile of livers from 14 healthy lean individuals, 12 obese individuals without liver disease, 15 individuals with biopsy-proven NAFLD and 16 individuals with biopsy-proven NASH^26^. We reanalyzed their data (38,732 total SE) to identify changes in RNA splicing between the four groups (Table S19) and compared the splicing events to SRSF3-dependent events derived from HepG2 cells (Fig. 6A). SRSF3-dependent SE were almost twofold enriched in the obese group compared to the lean healthy group (OR 1.88, p = 0.0003), but were very significantly depleted in NAFLD and NASH groups (OR 0.19, p < 0.0001 and OR 0.00, p < 0.0001, respectively). Hoang et al. profiled 78 individuals with varying degrees of biopsy-proven NAFLD and stratified them by steatosis, fibrosis, ballooning, and inflammation scores^24^. We analyzed this dataset (80,172 total SE) for changes in SEs with each of these scores (Table S20) and compared to the SRSF3-dependent SEs from HepG2 cells. SEs that were altered with steatosis or ballooning were significantly enriched for SRSF3-dependent SEs (OR 1.43, p = 0.0018; OR 1.31, p = 0.021; respectively) but were not enriched when analyzed by fibrosis or inflammation scores (Fig. 6B). In the largest study, Govaere et al. analyzed 206 individuals with liver disease (51 NAFLD, 155 NASH) and 10 healthy controls^25^. SEs that were significantly altered (out of 85,026 total SE) in the comparison between groups (Table S21) or between NAS scores were enriched for SRSF3-dependent SEs (OR 1.55, p < 0.0001 and OR 1.38, p = 0.0007, respectively) but no enrichment was seen based on fibrosis scores (Fig. 6C). In all three studies, enrichment of SRSF3-dependent SEs was seen with NAFLD (NAS, steatosis, ballooning) but not in NASH (fibrosis, inflammation) suggesting that changes in SRSF3-dependent splicing occur early in liver disease. We then compared the significant SEs between the four studies (Fig. 6D). Of the 30,436 SEs that were detected in the four datasets, only 9 SEs were common to all four studies, but 136 were significantly altered in at least three studies. For many of these, the SE did not quite reach significance for one out of the four studies. For example, alternative splicing of an upstream exon in the TPM1 gene was significantly increased in three datasets but did not quite reach significance in the Suppli dataset (p = 0.053), whereas that in the PANK1 did not reach significance in the Govaere dataset (p = 0.055) (Fig. S5A and B). In contrast, alternative splicing of a mutually exclusive exon in the IRF3 gene was significant in three datasets but was not detected in our dataset (Fig. S5C).

The differences in SEs between the groups, especially those with more advanced liver disease, could be explained by the presence of infiltrating immune cells or activation of Kupffer or hepatic stellate cells. So, we analyzed the tissue or cell of origin of the genes harboring the 136 common SE changes. The greatest enrichment was for SEs in genes that were specific to liver tissue, HepG2 cells or liver cells (Fig. 6E). We also observed enrichment of genes found in adipocytes, which would be consistent with the steatosis. The cell type signatures were primarily derived from hepatocytes (Fig. S5D). We confirmed this conclusion by analyzing the transcription factors that regulate expression of the genes harboring the 136 SEs. The top four enriched transcription factors based on TTRUST^50^ are liver specific (NR1H4/FXR, HNF4A, CEBPA, NR1I2/PXR) (Fig. 6F) and the top transcription factor targets based on GTRD ChIP-seq data are GTF2A2, TAF9B, ZNF318, and NR1H4/FXR (Fig. 6G). To assess which pathways or processes might be affected by the changes in SE, the genes containing the 136 common SEs were mapped to gene ontologies then clusters of significant GO terms and their interactions were plotted as a network (Fig. 6H). This analysis highlighted a large network of GO clusters representing metabolic functions of the liver, including complement, platelet degranulation, acute phase response, xenobiotic and drug metabolism, nuclear receptor and FXR pathways, and NAFLD (Fig. 6H). There were also subclusters centered on mRNA transport and translation. Mapping the SEs to DisGeNet highlighted chronic liver disease, liver injury, hepatitis, and hypoglycemia among the major disease association (Fig. S5E). From this we conclude that the SEs common to multiple studies are likely derived from hepatocytes rather than infiltrating immune cells.

### Early versus late disease changes in alternative splicing

**Figure 7:**
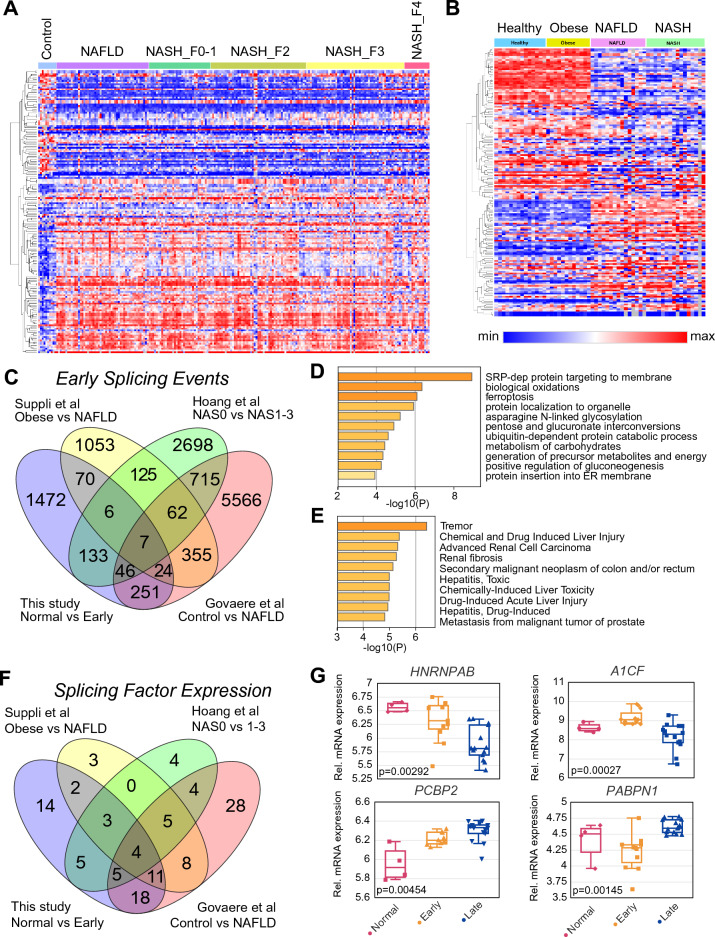
Changes in RNA splicing in early disease. (**A**) Heatmap showing top SEs in the Govaere et al. dataset clustered according to group. SEs cluster into two groups which either have increased or decreased PSI going from healthy controls to NAFLD. Red indicates high PSI, blue low PSI. (**B**) Heatmap showing top SEs in the Suppli et al. dataset clustered according to group. SEs cluster into two groups which either have increased or decreased PSI going from Obese to NAFLD. (**C**) Overlap of early SEs from the four studies based on a Kruskal p < 0.05 threshold. The groups compared are shown for each study. (**D**) and (**E**) Pathway and disease enrichment of genes harboring 145 early SEs common to 3 or more studies. (**F**) Overlap of changes in splicing factor expression during early disease in the four studies. Splicing factors analyzed were taken from Sebestyén et al.^86^. (**G**) Relative expression of the four splicing factors (*HNRNPAB, A1CF, PCBP2* and *PABPN1*) that are altered in all four datasets in normal early or late clusters in our study. Statistical significance is for Cluster A vs Cluster B.

As the previous analysis indicated that SRSF3-dependent SEs were more enriched with steatosis rather than with fibrosis or inflammation, we created heatmaps for the three additional datasets based on the top 500 SEs. For each dataset, the SEs were further filtered to remove SEs found in less than half the healthy control samples, or with PSI variances less than 0.001 (0.1% of max PSI). Visual inspection of the heatmaps shows that there is a major change in splicing in the transition from control to NAFLD in the Govaere dataset (Fig. 7A), from obese to NAFLD in the Suppli dataset (Fig. 7B), or from NAS 0 to NAS 1 in the Hoang dataset (Fig. S6A). We then focused on these early changes in splicing by comparing the SEs altered in these transitions (Fig. 7C). Only 145 early SEs were significantly altered in three or more datasets, and these showed enrichment for genes involved in carbohydrate metabolism, gluconeogenesis and glycogen synthesis, protein localization to organelles and membranes, and autophagy and ferroptosis (Fig. 7D) and were also associated with acute drug- or chemical-induced liver injury and hepatitis, as well as fibrosis and malignant neoplasms (Fig. 7E). To compare the common early SE changes to the total altered early SEs in the four individual datasets, we mapped all the early SEs for each dataset (FDR-adjusted) to GO biological processes. The Circos plot shows the overlap of the genes harboring the SEs based on shared functions or pathways (Fig. S6B). Mapping to GO terms showed enrichment of vesicle and Golgi transport, cellular responses to stress, and lipid metabolism in all four datasets and three of the datasets showed enrichment of small molecule metabolism (Fig. S6C). These results suggest that these early splicing alterations are involved in normal liver function and response to injury.

To understand which splicing factors might be driving these changes, we then analyzed expression of splicing factors and RNA-binding proteins during the early obese to NAFLD transition (Fig. 7F). Only four splicing factors, *A1CF, HNRNPAB, PABPN1* and *PCBP2* were altered in early disease in our data (Fig. 7G) and the other three datasets (Fig. S6D–F). An additional 24 splicing factors (*PCBP1, SFPQ, SRSF8, KHSRP, ESRP2, HNRNPH2, KHDRBS1* (Sam68)*, CELF2, ZCRB1, HNRNPUL1, RBFOX2, RBMS1, MATR3, RBM8A, RBM7, RBM38, HNRNPD, CPEB3, SART3, ZC3H14, CELF1, HNRNPM, CNOT4* and *SRRM2*) were altered in three out of four datasets.

**Figure 8:**
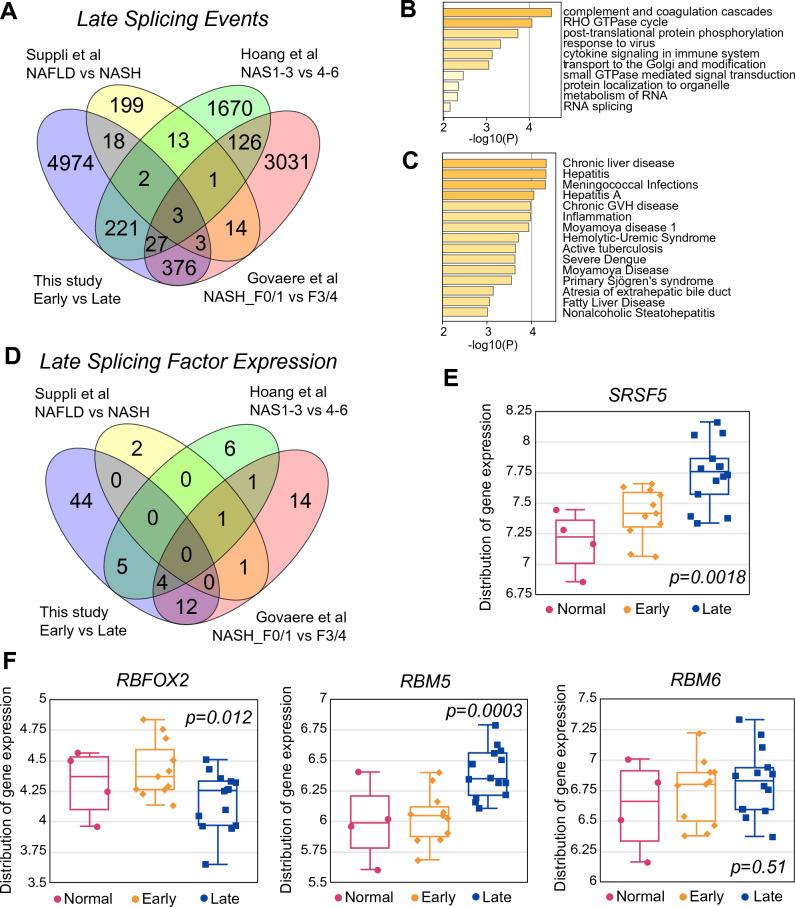
Changes in RNA splicing in late disease. (**A**) Overlap of late SEs from the four studies. The groups compared are shown for each study. (**B**) and (**C**) Pathway and disease enrichment of genes harboring 36 late SEs common to 3 or more studies. (**D**) Overlap of changes in splicing factor expression during late disease in the four studies. Splicing factors analyzed were taken from Sebestyén et al.^86^. (**E**) and (**F**) Relative expression of the four splicing factors (*SRSF5, RBFOX2, RBM5* and *RBM6*) that are altered in all four datasets in the normal, early or late clusters in our study. Statistical significance is for early vs late clusters.

We also analyzed the late changes in SEs that occur during the NASH progression with increased inflammation and fibrosis; NASH_F0/1 vs NASH_F3/4 in the Govaere dataset, NAFLD vs NASH in the Suppli dataset, or NAS 1–3 vs NAS 4–6 in the Hoang dataset. Thirty-six SEs were altered in three out of four datasets (Fig. 8A). The 36 genes harboring the common late SEs show enrichment in immune response genes and RHO GTPase signaling (Fig. 8B) that is not observed in the early SE changes and are associated with chronic liver disease, hepatitis, fatty liver disease, NASH, obesity, and many inflammatory and infectious diseases (Fig. 8C). Comparing all the late SEs in the four individual datasets, the Circos plot again shows extensive overlap (Fig. S7A) and the enriched terms now include chromatin and cytoskeletal organization and DNA damage in addition to the previous metabolic terms (Fig. S7B). These results suggest that the late splicing alterations are also involved in cytoskeletal and genomic changes, and DNA damage, indicative of more advanced disease. There were no splicing factors that were altered in all four late datasets but *HNRNPAB, RBM6, RBM5, SRSF5* and *RBFOX2* were altered in three out of four (Fig. 8D). *HNRNPAB* and *RBFOX2* were the only genes whose expression was altered in both early and late disease*,* and *RBM6, RBM5* and *SRSF5* were only altered in late disease (our data in Figs. 7F and 8E,F, other datasets in Fig. S7C–E).

### Changes in alternative splicing in hepatocellular carcinoma

**Figure 9:**
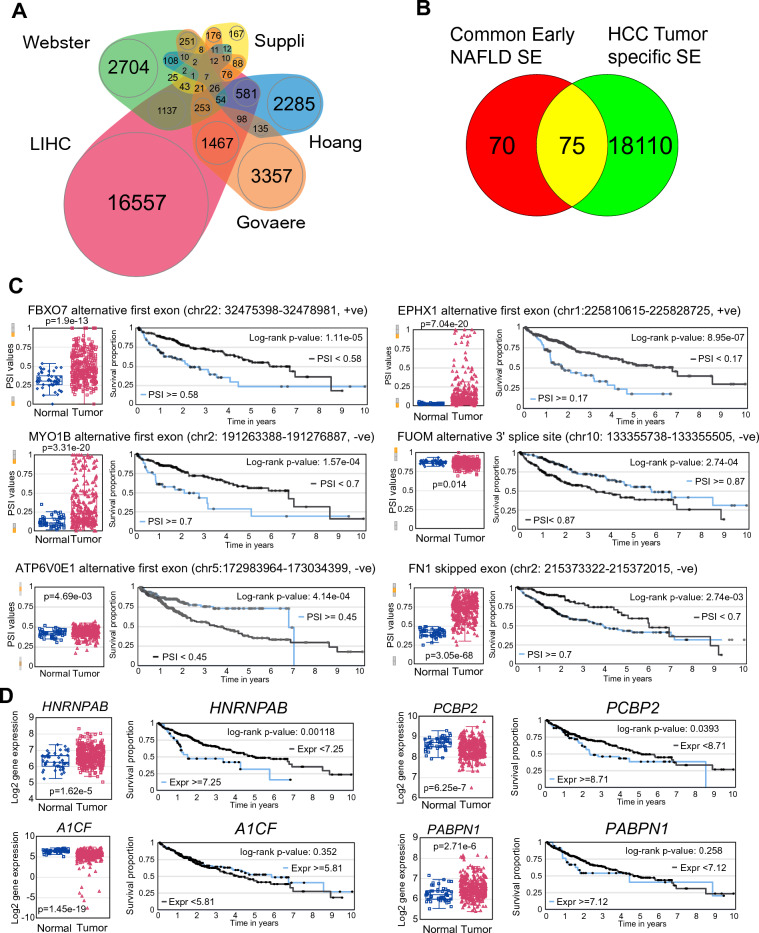
Altered RNA splicing events in early metabolic liver disease are conserved in HCC. (**A**) Overlap of total SEs in the four liver datasets with changes in splicing in the TCGA HCC data (LIHC). (**B**) Overlap of the 145 common early SEs with changes in splicing in HCC data. (**C**) Six of the top common early SEs that correlate with survival. Box plot shows individual PSI values for normal liver and tumor in the HCC data. Survival curves are based on optimal PSI values for each SE. Log-rank p-value is shown. (**D**) Expression of the four common early splicing factors in the HCC data. Box plot shows individual log2 expression values for normal liver and tumor. Survival curves are based on optimal expression cut-off for each gene. Log-rank p-value is shown.

The changes in RNA splicing events that we identified occurred mainly during early liver disease (obesity to simple steatosis), so we then investigated whether any of the early changes would persist in hepatocellular carcinoma. Analysis of the TCGA LIHC dataset^51^ using the same approach identified 159,714 splicing events of which 18,118 were altered (Kruskal p < 0.05) in tumors compared to normal adjacent tissue (Table S22). These SEs were compared to the significant SEs identified for each of the four NAFLD datasets (Fig. 9A). Thirty percent of the significant SEs from this study and Govaere et al. were also significantly altered in the LIHC data compared to 55% of the SEs from Suppli et al. and Hoang et al., indicating that many of these NASH-associated SEs are still present in HCC. Overall, 22% of the tumor-associated SEs were found in the NASH-associated SEs compared to 12% of total SEs, hence tumor-associated SEs were significantly enriched for NASH-associated SEs (OR 2.13, p < 0.0001). Looking more closely at early changes, we mapped the common 145 early SEs that were found in three or more NAFLD datasets to the HCC data; 75 out of the 145 SEs (52%) were significantly altered in the tumors in the TCGA LIHC dataset (Fig. 9B) compared to only 22% for other SEs (OR = 3.9, χ^2^ p < 0.0001). We calculated the survival log-rank p-value for each of these early SEs based on the optimal PSI cut-off. Twenty-eight SEs (37%) out of the 75 SEs were significantly associated with survival compared to only 26% of other tumor-associated SEs. Of the 28 SEs that are associated with survival, 23 SEs cause alterations in the protein sequence (Table S23). Six of the top early NAFLD-associated SEs that are conserved in HCC and associated with survival are (i) an alternative first exon that deletes a ubiquitin-like domain in the FBXO7 gene, an F-box containing protein that interacts with the ubiquitin ligase complex SCF, (ii) an alternative first exon in the EPHX1 gene that encodes epoxide hydrolase 1, (iii) an alternative first exon on the myosin-1b (MYO1B) mRNA that leads to the production of a protein containing only the myosin head domain, (iv) an alternative 3′ splice site in the first exon of the FUOM fucose mutarotase protein, (v) an alternative first exon in the ATP6V0E1 gene that deletes a transmembrane helix, and (vi) a profibrogenic cassette exon in the fibronectin 1 (FN1) gene (Fig. 9C).

We also looked at expression of the splicing factors that were altered in the NAFLD datasets in the HCC. The four splicing factors *HNRNPAB*, *PCBP2, A1CF* and *PABPN1,* which were altered in all four NAFLD datasets, also have altered expression in HCC tumors but only *HNRNPAB* and *PCBP2* are associated with survival (Fig. 9D). We extended this analysis to splicing factors conserved in three out of four datasets (Fig. S8). For ten splicing factors, *SFPQ, KHDRBS1, HNRNPM, HNRNPUL1, RBMS1, MATR3, RBM8A, SRSF8, SART3* and *ZCRB1,* higher expression was associated with poorer survival (Fig. S8A) whereas for six splicing factors, *CELF2, CPEB3, SRSF5, RBFOX2, ESRP2,* and *SRRM2,* higher expression was associated with better survival (Fig. S8B). Expression of *PCBP1, KHSRP, HNRNPH2, RBM7, RBM38, HNRNPD, ZC3H14, CELF1, CNOT4, RBM5,* or *RBM6* was not associated with survival, independent of any change in expression in HCC. These results suggest that many early alterations in RNA splicing are conserved through disease progression and can influence survival.

## Discussion

In this paper, we analyzed changes in RNA splicing in 29 samples of liver tissue from individuals with non-alcoholic fatty liver disease and found extensive changes in alternative splicing. We compared the splicing results to a parallel transcriptome analysis and found that while the transcriptome data showed good separation of individuals with NASH from those with NAFLD or normal individuals, there was no separation of normal versus NAFLD. The splicing analysis on the other hand allowed separation of the normal and NAFLD samples in addition to separating from the NASH samples. We demonstrated alterations in RNA splicing using three independent bioinformatic approaches and to test the generality of our findings, we confirmed changes in RNA splicing in three large liver transcriptome datasets. Most of the splicing changes in these larger datasets occurred with the transition to steatosis, with fewer changes occurring with the onset of fibrosis and inflammation. As might be expected, common splicing events that are altered early in liver disease are associated with metabolic pathways such as lipid and carbohydrate metabolism and acute liver injury, whereas those in later disease also show enrichment of chronic liver injury and inflammation, DNA damage and cytoskeletal reorganization. The changes in RNA splicing during early liver disease were significantly enriched for targets of the splicing factor SRSF3, which is consistent with our previously published finding that the SRSF3 protein is degraded during lipid overload or in NAFLD in both human and mouse samples^29^.

In contrast, the late splicing changes that occurred with inflammation or fibrosis were not enriched for SRSF3 targets. The SRSF3 targets, however, only represented a small fraction of the total changes in splicing so other splicing factors might be involved and, indeed, we found that many RNA-binding proteins showed altered splicing in the NAFLD and NASH samples suggesting multifactorial dysregulation of RNA splicing. This conclusion is supported by other studies showing dysregulation of components of the splicing apparatus in NAFLD/NASH^1,11,33^. The majority of these studies analyzed RNA expression by microarray or RNAseq but interpretation of the results is difficult as many RNA binding proteins autoregulate their own expression through inclusion of poison exons^52–54^. Studies that evaluated splicing factor protein expression are far fewer, but Wang et al. observed the upregulation of NONO, SRSF6, HNRNPA2/B1, HNRNPH and confirmed the downregulation of SRSF3 protein in livers of mice with NAFLD^33^. Indeed, DRAK2 prevents nuclear localization of SRSF6, altering RNA splicing and aggravating NAFLD^55^.

Many of the observed changes in splicing in early NAFLD/NASH are also observed in HCC and associated with survival. That these changes are maintained throughout the oncogenic process suggests that they may contribute to cancer development or support tumor growth. A limitation of this study is that it is difficult to predict the effect of altered splicing events on cellular or tissue phenotypes as the splicing events in most cases do not uniquely define the transcript isoform, and most alternative transcript isoforms have no functional annotation. Transcript isoform ratios can be estimated by deconvoluting the data but that only generates known isoforms and cannot predict novel transcript isoforms. Furthermore, without knowledge of the function of the protein isoforms it is not possible to predict functional changes. Given these limitations, interpretation is limited to assessing the functional roles of the genes that harbor the splicing events without knowing whether an event increases or decrease gene function. Nevertheless, the top NAFLD-associated splicing events that were conserved in HCC and correlated with survival have been implicated in either cellular proliferation or cancer. FBXO7 gene regulates ubiquitin-dependent CyclinD/CDK6 degradation and maintains mesenchymal gene expression^56,57^ and mutations have been associated with Parkinson's disease but it's role in the liver has not been determined. EPHX1 is downregulated in HCC and reduces tumorigenicity in mice but may contribute to chemoresistance^58^. FUOM isomerizes α- and β-fucose for N- and O-linked fucosylation that is increased in HCC^59^. Myosin-1b (MYO1B) promotes cancer by targeting HIF1a and SNAI2/cyclinD1^60,61^. ATP6V0E1 encodes the E subunit of the vacuolar ATPase that is important for cancer cell survival in pancreatic cancer^62^. Lastly, Fibronectin 1 (FN1) can have positive or negative effects on cancer growth depending on the tissue^63^.

The observed changes in splicing factor expression are also maintained in HCC and are associated with survival. Some of these have documented roles in HCC development. For example, A1CF promotes NASH and HCC^64^, ESRP2 loss promotes HCC and lower expression predicts worse survival^32^, HNRNPAB induces EMT and promotes HCC metastasis^65^, the poly(C)-binding protein PCBP2 is upregulated in HCC and promotes proliferation^66^, SFPQ and RBM8A mediate resistance to platinum drugs in HCC^67,68^, RBMS1 blocks ferroptosis in HCC^69^, MATR3 promotes HCC progression^70^, SART3 is an immunotherapy target in HCC^71^, ZCRB1 is a signature gene for HCC^72^, and down regulation of CELF2 or CPEB3 promotes HCC^73^, but the targets for many of these splicing factors remain to be determined. The role of these splicing factors in early liver disease is less clear but mouse studies have highlighted a potential causative role for some splicing factors in the pathogenesis of NAFLD and NASH. KHDRBS1 (Sam68) and SRSF3 regulate gluconeogenesis^9^ and loss of SRSF3, A1CF, SRSF2, SRSF1, and SRSF10 causes steatosis and changes in lipid metabolism^74^ but loss of NONO, the binding partner for SFPQ, increases fat catabolism and protects against steatosis^75^. Splicing factors are also involved in liver maturation as loss of SRSF3, SRSF7, ESRP2 or RBM15 reduces hepatocyte differentiation^9,32,76,77^. Indeed, many of these factors can have opposing effects. PCBP2 overcomes palmitate-induced insulin resistance in HepG2 cells via inhibition of HIF1a and STAT3^78^ but, in more advanced disease, PCBP2 promotes collagen production and liver fibrosis^79^. The contribution of each of these splicing factors to the dysregulation of RNA splicing in human NAFLD and NASH, however, remains to be determined. An alternative approach is to assess the splicing of known target genes but that requires identification of direct targets through binding and knockout studies. We have shown that known SRSF3 target genes are altered in NAFLD, NASH and HCC suggesting that SRSF3 splicing function is reduced but that remains to be demonstrated for other factors.

In summary, dysregulated RNA splicing is observed early in metabolic liver disease and many of these changes are maintained during disease progression and are even found in HCC. The fact that the altered splicing is maintained through a long disease progression over many years argues for their importance in the disease pathogenesis. While the function of the individual transcriptional isoforms is not known for most splicing events, the genes themselves have documented roles in liver metabolism, cell proliferation and tumorigenesis supporting the importance of these events. Further studies investigating individual mRNA isoforms and the proteins produced will be required for a full understanding of the disease process.

## Materials and methods

### Human tissue samples

Human liver samples were obtained from the Liver Tissue and Cell Distribution Service at the University Minnesota, the Department of Pathology at the VASDHS, and the Moores Cancer Center Biorepository**.** Subject characteristics are provided in Supplemental Table 1. Liver and other tissues were harvested at sacrifice for both histology and RNA extraction. Livers were fixed in formalin for 24 h followed by washing in 70% ethanol. Paraffin embedded sections (5 μm) were cut, dewaxed, and stained with hematoxylin and eosin. All human studies were approved by the UCSD Institutional Review Board.

### Isolation of murine primary hepatocytes

Primary hepatocytes were obtained by two-step perfusion with liver perfusion medium, SC-1 (137 mM NaCl, 5.4 mM KCl, 0.56 mM NaH_2_PO_4_⋅H_2_O, 0.85 mM Na_2_HPO_4_, 10 mM HEPES, 4.2 mM NaHCO_3_, 0.5 mM EGTA, 5 mM Glucose) followed by digestion medium, SC-2 (137 mM NaCl, 5.4 mM KCl, 0.56 mM NaH_2_PO_4_⋅H_2_O, 0.85 mM Na_2_HPO_4_, 10 mM HEPES, 4.2 mM NaHCO_3_ 12 mM CaCl_2_⋅H_2_O) containing 0.5 mg/mL collagenase D (Roche, Indianapolis, IN). Liver cells were disaggregated by passing through a 100 μm pore nylon mesh Cell Strainer (BD Biosciences, San Jose, CA) and centrifuged at 100 × RCF for 10 min at 4 °C. Cells pellets were suspended in 36% percoll and centrifuged at 60 × RCF for 6 min. The subsequent cell pellets were washed with SC-2 buffer without collagenase-D, the numbers of total viable cells were determined by Trypan blue staining, and cells were plated on collagen-coated plates (Invitrogen, Carlsbad, CA). Primary hepatocyte cells were cultured in William E culture medium supplemented with 10% fetal bovine serum, and 100 U mL^−1^ of penicillin and 100 μg mL^−1^ of streptomycin at 37 °C. All animal studies were approved by the UCSD Institutional Animal Care and Use Committee.

### RNAseq analysis

RNAseq was performed on RNA from frozen human liver samples and on RNA from primary mouse hepatocytes with adenoviral-mediated acute loss of SRSF3 using an Illumina Nextgen short-read sequencer (paired-end 100). Raw reads were trimmed and QC’d. Sequencing reads from HepG2 cells with shRNA knockdown of SRSF3 or control shRNA were downloaded from ENCODE (ENCSR376FGR). Fastq reads were aligned to the human (hg38) or mouse (mm10) genomes using the STAR-2pass method^80^ then alternative splicing (AS) events identified using the percent spliced in (PSI) metric with the Psichomics software in R^34^. STAR gene counts were normalized using trimmed mean of M-values (TMM), log-transformed, and IDs converted to gene symbols. STAR splice-junction counts were converted to the percent-spliced in metric (PSI) and annotated using human hg38. Principal component analysis (PCA) was performed on PSI and gene expression data. Many splicing events were either not detected or had fewer than 10 reads, so dimension reduction was performed by filtering out events with more than 5 missing values. Non-parametric tests (Wilcoxon or Kruskal–Wallis rank sum) were used to assess differential splicing as the percent spliced in (PSI) data are not normally distributed. A p-value of 0.05 was used to identify potential differential AS events which were further refined using an FDR of 0.05. Data were also analyzed by MAJIQ^40^ and Whippet^41^ using default parameters. Mouse genes showing HFD-induced AS events were compared to genes with SRSF3-dependent AS events to identify SRSF3-dependent changes in mouse NAFLD, and genes with SRSF3-dependent splicing events were mapped to human homologs to allow comparison with the human NAFLD data. Heatmaps were generated using Morpheus (https://software.broadinstitute.org/morpheus). Immune and liver cell composition was analyzed using CIBERSORTx^44^ using the Human Liver Cell Atlas^45^ or the Bader Human Liver Atlas^43^. Enrichment analysis and protein-interaction network generation was performed using Metascape^36^.

### SRSF3-seCLIP analysis

Primary mouse hepatocytes were isolated and subjected to seCLIP following published procedures by Eclipse Biosciences^46,81^ (La Jolla, CA). SRSF3 was precipitated using mouse monoclonal 7B4 anti-SRSF3 antibody (1:100 dilution, ATCC CRL-2384, Manassas, VA). CLIP reads were aligned to the mouse mm10 genome then peaks of SRSF3 binding were identified using the HOMER suite^82^ or using Pureclip^47^. Sequences enriched at the crosslink sites identified by Pureclip were analyzed using DREME^48^ to identify conserved motifs and FIMO^49^ to find individual occurrences. Alternative splicing events and SRSF3 binding were co-visualized on individual genes using IGV^83^. Correlation of SRSF3 binding peaks and alternative splicing events was performed using custom scripts in R. Splicing events were called as SRSF3-dependent if they were altered in the SRSF3 knockout hepatocytes with p < 0.05 and contained a SRSF3 binding peak that mapped to < 1000 nt from the splicing event.

### TCGA liver cancer data analysis (LIHC)

HCC gene counts, exon-junction counts, and metadata files were downloaded from the TCGA website. The STAR hg38-aligned TCGA gene count, splice junction and clinical data were downloaded and formatted for the Psichomics program. Gene counts and splice-junction counts were imported and annotated using human hg38 as above. Individual gene expression or alternative splicing events were visualized on violin plots. Survival analysis was performed using selected gene expression or PSI cutoffs, generating Kaplan–Meier curves and deriving Cox proportional hazard models. Correlation analysis between gene expression and an alternative splicing event was performed using Spearman rank correlation.

### Immunoblot analysis

Equal amounts of cellular protein (10 μg) were separated by SDS-PAGE using 4–15% or 20% Criterion precast polyacrylamide gels (Bio-Rad), transferred to PVDF membranes (MilliporeSigma, Burlington, MA), blocked with 5% BSA for 1 h at RT and immunoblotted with primary antibodies overnight at 4 °C followed by HRP-conjugated secondary antibodies at room temperature for 1 h, washed 3× in TBS-Tween20 then developed using a chemiluminescent substrate kit (Pierce, Rockford, IL). Antibodies used for immunoblotting were mouse monoclonal 7B4 anti-SRSF3 antibody (1:1000 dilution, ATCC CRL-2384, Manassas, VA), HRP labeled anti-mouse (sc516102) secondary antibody (1:5000 dilution, Santa Cruz Biotechnology, Santa Cruz, CA). Blots were quantified using a Gel-Doc imaging system (Bio-Rad).

### Statistical analysis

Data was analyzed by 1-way or 2-way ANOVA followed by Tukey multiple comparison post-test, or Students’ t-test as appropriate using Prism (Graph Pad, La Jolla, CA) or in R statistical software (v3.4.4). Normality was assessed by D’Agostino-Pearson omnibus normality test. Results were expressed as mean ± standard error and considered significant with p < 0.05. Linear regression and Chi-squared contingency table analysis was performed using Prism. Venn diagrams were generated using Venny^84^ or nVenn^85^.

### Supplementary Information

Supplementary Figures.

Supplementary Tables.

## Data Availability

Supporting Information is available from the author. All the data supporting the findings of this study are available within the article and its supplementary information files and from the corresponding author upon reasonable request. Sequence reads are available from the Sequence Read Archive (SRA BioProject ID PRJNA1040226). A reporting summary for this article is available as a Supplementary Information file.
