# Supplementary material for: Dysregulation of RNA splicing in early non-alcoholic fatty liver disease through hepatocellular carcinoma
Source: Sci Rep. 2024 Jan 30;14:2500. doi: 10.1038/s41598-024-52237-7 (PMC10828381; doi:10.1038/s41598-024-52237-7)
Supplement: Supplementary file 1 — Supplementary Figures. [file 41598_2024_52237_MOESM1_ESM.pdf]

Supplemental Figures:

Supplemental Figure 1: RNA splicing is altered in NAFLD and NASH analyzed by Psichomics.

Supplemental Figure 2: Metascape analysis of alternative splicing analyzed by MAJIQ or Whippet.

Supplemental Figure 3: Transcriptome analysis of NAFLD and NASH samples.

Supplemental Figure 4: Fatty acid treatment reduces SRSF3 protein levels in HepG2 cells.

Supplemental figure 5: Comparison of selected splicing events across four datasets.

Supplemental Figure 6: Early changes in splicing.

Supplemental Figure 7: Late changes in splicing.

Supplemental Figure 8: Survival curves for common splicing factors.

Supplemental Figure 9: Original blots for Supplemental Figure 4

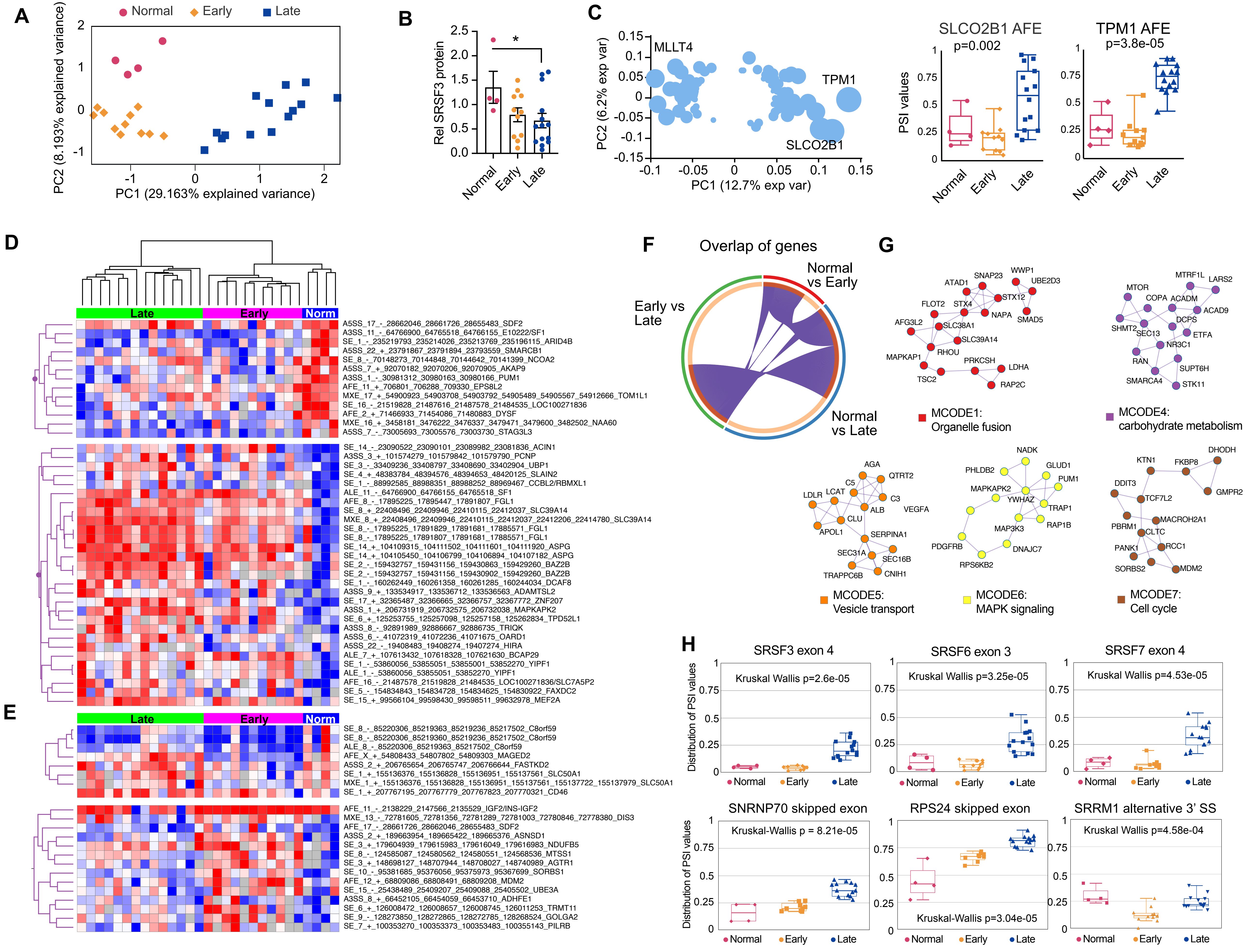

A
Normal
Early
Late
PC2 (8.193% explained variance)
PC1 (29.163% explained variance)
B
Rel SRSF3 protein
Normal
Early
Late
C
PC2 (6.2% exp var)
PC1 (12.7% exp var)
MLLT4
TPM1
SLCO2B1
SLCO2B1 AFE
p=0.002
TPM1 AFE
p=3.8e-05
PSI values
Normal
Early
Late
D
Late
Early
Norm
A5SS_17_-_28662046_28661726_28655483_SDF2
A3SS_11_-_64765900_64765518_64766155_E10222/SF1
SE_1_-_235219793_235214026_235213769_235196115_ARID4B
A5SS_22_+_23791867_23791894_23793559_SMARCB1
SE_8_-_70148273_70144848_70144642_70141399_NCOA2
A5SS_7_+_92070182_92070206_92070905_AKAP9
A3SS_1_-_30981312_30980163_30980166_PUM1
AFE_11_+_706801_706288_709330_EPS8L2
MXE_17_+_54900923_54903708_54903792_54905489_54905567_54912666_TOM1L1
SE_16_-_21519828_21487616_21487578_21484535_LOC100271836
AFE_2_+_71466933_71454086_71480883_DYSF
MXE_16_+_3458181_3476222_3476337_3479471_3479600_3482502_NAA60
A5SS_7_-_73005693_73005576_73003730_STAG3L3
SE_14_-_23090522_23090101_23089982_23081836_ACIN1
A3SS_3_+_101574279_101579842_101579790_PCNP
SE_3_-_33409236_33408797_33408690_33402904_UBP1
SE_4_+_48383784_48394576_48394653_48420125_SLAIN2
SE_1_-_88992585_88988351_88988252_88969467_CCBL2/RBMXL1
ALE_11_-_64766900_64766155_64765518_SF1
AFE_8_-_17895225_17895447_17891807_FGL1
SE_8_+_22408496_22409946_22410115_22412037_SLC39A14
MXE_8_+_22408496_22409946_22410115_22412037_22412206_22414780_SLC39A14
SE_8_-_17895225_17891829_17891681_17885571_FGL1
SE_8_-_17895225_17891807_17891681_17885571_FGL1
SE_14_+_104109315_104111502_104111601_104111920_ASPG
SE_14_+_104105450_104106799_104106894_104107182_ASPG
SE_2_-_159432757_159431156_159430863_159429260_BAZ2B
SE_2_-_159432757_159431156_159430902_159429260_BAZ2B
SE_1_-_160262449_160261358_160261285_160244034_DCAF8
A3SS_9_+_133534917_133536712_133536563_ADAMTSL2
SE_17_+_32365487_32366665_32366757_32367772_ZNF207
A3SS_1_+_206731919_206732575_206732038_MAPKAPK2
SE_6_+_125253755_125257098_125257158_125262834_TPD52L1
A3SS_8_-_92891989_92886667_92886735_TRIQK
A5SS_6_-_41072319_41072236_41071675_OARD1
A5SS_22_-_19408483_19408274_19407274_HIRA
ALE_7_+_107613432_107618328_107621630_BCAP29
SE_1_-_53860056_53855051_53855001_53852270_YIPF1
ALE_1_-_53860056_53855051_53852270_YIPF1
AFE_16_-_21487578_21519828_21484535_LOC100271836/SLC7A5P2
SE_5_-_154834843_154834728_154834625_154830922_FAXDC2
SE_15_+_99566104_99598430_99598511_99632978_MEF2A
E
Late
Early
Norm
SE_8_-_85220306_85219363_85219236_85217502_C8orf59
SE_8_-_85220306_85219360_85219236_85217502_C8orf59
ALE_8_-_85220306_85219363_85217502_C8orf59
AFE_X_+_54808433_54807802_54809303_MAGED2
A5SS_2_+_206765654_206765747_206766644_FASTKD2
SE_1_+_155136376_155136828_155136951_155137561_SLC50A1
MXE_1_+_155136376_155136828_155136951_155137561_155137722_155137979_SLC50A1
SE_1_+_207767195_207767779_207767823_207770321_CD46
AFE_11_-_2138229_2147566_2135529_IGF2/INS-IGF2
MXE_13_-_72781605_72781289_72781003_72780846_72778380_DIS3
AFE_17_-_28661726_28662046_28655483_SDF2
A3SS_2_+_189663954_189665422_189665376_ASNSD1
SE_3_+_179604939_179615983_179616049_179616983_NDUFB5
SE_8_-_124585087_124580562_124580551_124568536_MTSS1
SE_3_+_148698127_148707944_148708027_148740989_AGTR1
SE_10_-_95381685_95376056_95375973_95367699_SORBS1
AFE_12_+_68809086_68808491_68809208_MDM2
SE_15_-_25438489_25409207_25409088_25405502_UBE3A
A3SS_8_+_66452105_66454059_66453710_ADHFE1
SE_6_+_126008472_126008657_126008745_126011253_TRMT11
SE_9_-_128273850_128272865_128272785_128268524_GOLGA2
SE_7_+_100353270_100353373_100353483_100355143_PILRB
F
Overlap of genes
Normal vs Early
Early vs Late
Normal vs Late
G
MCODE1: Organelle fusion
MCODE4: carbohydrate metabolism
MCODE5: Vesicle transport
MCODE6: MAPK signaling
MCODE7: Cell cycle
H
SRSF3 exon 4
Kruskal Wallis p=2.6e-05
SRSF6 exon 3
Kruskal Wallis p=3.25e-05
SRSF7 exon 4
Kruskal Wallis p=4.53e-05
SNRNP70 skipped exon
Kruskal-Wallis p = 8.21e-05
RPS24 skipped exon
Kruskal-Wallis p=3.04e-05
SRRM1 alternative 3' SS
Kruskal Wallis p=4.58e-04
Distribution of PSI values
Normal
Early
Late

Supplemental Figure 1: RNA splicing is altered in NAFLD and NASH analyzed by psichomics: A: PCA plot based on the analysis of alternative splicing events in RNA from 29 liver samples in three clusters after dimension reduction by filtering out splicing events with more than 5 missing values which reduced the dataset to 6,797 SEs. B: SRSF3 protein expression in each cluster quantified by western blot. C: Plot showing top 100 SEs that contribute the most to the PCA. Each SE is shown by a blue circle, the size of the circle denoting the magnitude of the contribution. PSI values for the two top SEs, SLCO2B1 AFE and TPM1 AFE are shown in column graphs for the three clusters. D: Exploded view of heatmap of the clusters of SEs that are altered in the Normal cluster rather than the Early or Late clusters. Red indicates high PSI and blue low PSI. E: Exploded view of heatmap of the clusters of SEs that are altered in the Early cluster rather than the Normal or Late clusters. F: Circos plot showing overlap of genes harboring the altered SEs in pairwise comparisons of the three clusters. Purple lines connect the same genes. G: MCODE protein interaction networks for genes harboring the altered SEs. Networks are colored individually and functionally annotated. Nodes are shown as circles, interactions as lines. H: PSI values for alternative splicing of poison exons in the SRSF3, SRSF6 and SRSF7 genes, and in the splicing-related proteins SNRNP70, RPS24, and SRRM1 according to cluster. Kruskal-Wallis pvalues are given for the three group comparison.

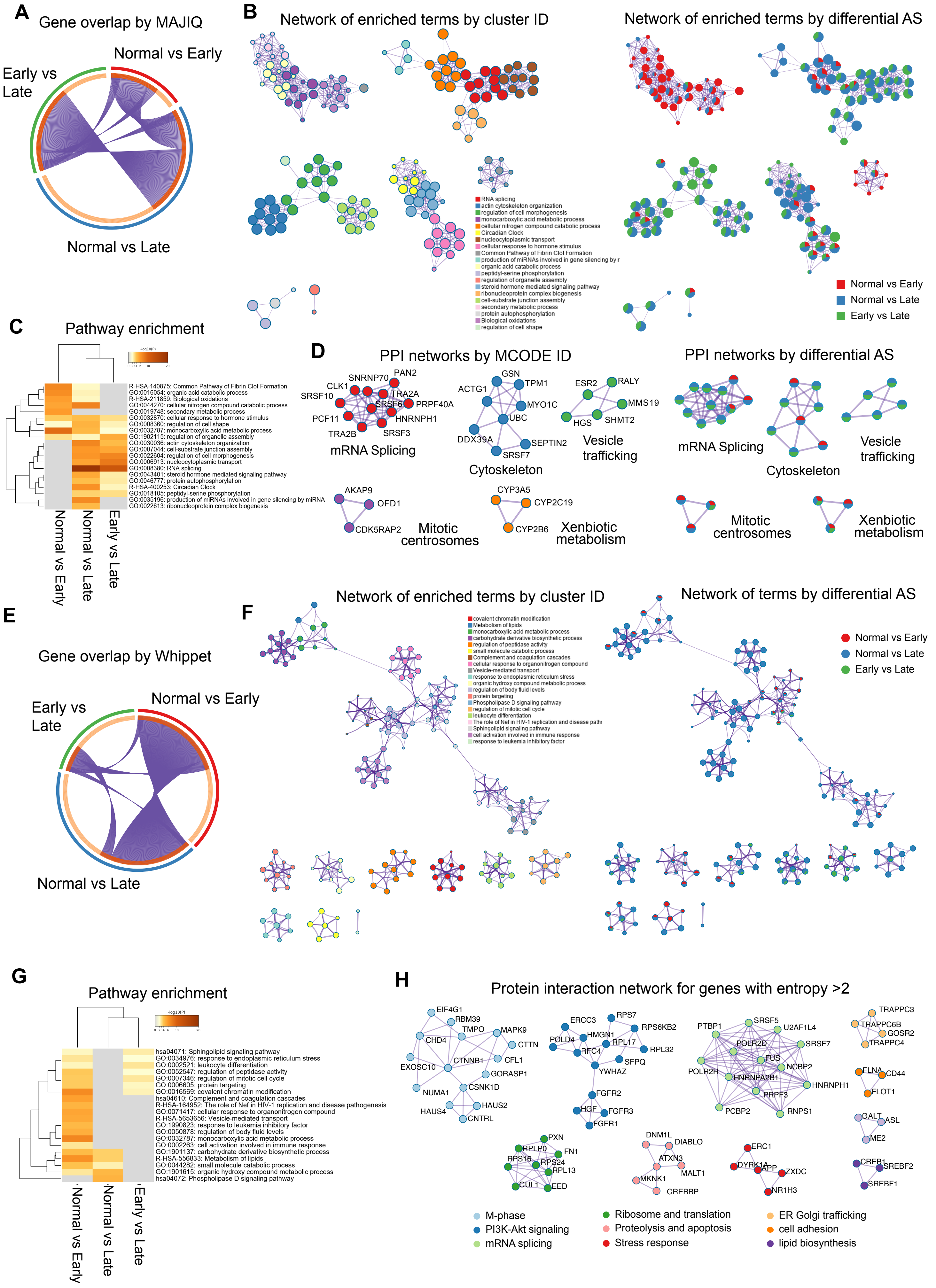
A
Gene overlap by MAJIQ
Normal vs Early
Early vs Late
Normal vs Late
B
Network of enriched terms by cluster ID
Network of enriched terms by differential AS
RNA splicing
actin cytoskeleton organization
regulation of cell morphogenesis
monocarboxylic acid metabolic process
cellular nitrogen compound catabolic process
Circadian Clock
nucleocytoplasmic transport
cellular response to hormone stimulus
Common Pathway of Fibrin Clot Formation
production of miRNAs involved in gene silencing by r
organic acid catabolic process
peptidyl-serine phosphorylation
regulation of organelle assembly
steroid hormone mediated signaling pathway
ribonucleoprotein complex biogenesis
cell-substrate junction assembly
secondary metabolic process
protein autophosphorylation
Biological oxidations
regulation of cell shape
Normal vs Early
Normal vs Late
Early vs Late
C
Pathway enrichment
-log10(P)
R-HSA-140875: Common Pathway of Fibrin Clot Formation
GO:0016054: organic acid catabolic process
R-HSA-211859: Biological oxidations
GO:0044270: cellular nitrogen compound catabolic process
GO:0019748: secondary metabolic process
GO:0032870: cellular response to hormone stimulus
GO:0008360: regulation of cell shape
GO:0032787: monocarboxylic acid metabolic process
GO:1902115: regulation of organelle assembly
GO:0030036: actin cytoskeleton organization
GO:0007044: cell-substrate junction assembly
GO:0022604: regulation of cell morphogenesis
GO:0006913: nucleocytoplasmic transport
GO:0008380: RNA splicing
GO:0043401: steroid hormone mediated signaling pathway
GO:0046777: protein autophosphorylation
R-HSA-400253: Circadian Clock
GO:0018105: peptidyl-serine phosphorylation
GO:0035196: production of miRNAs involved in gene silencing by miRNA
GO:0022613: ribonucleoprotein complex biogenesis
Normal vs Early
Normal vs Late
Early vs Late
D
PPI networks by MCODE ID
PPI networks by differential AS
SNRNP70
PAN2
CLK1
SRSF10
TRA2A
SRSF6
PRPF40A
PCF11
HNRNPH1
TRA2B
SRSF3
mRNA Splicing
ACTG1
GSN
TPM1
MYO1C
UBC
DDX39A
SEPTIN2
SRSF7
Cytoskeleton
ESR2
RALY
MMS19
HGS
SHMT2
Vesicle trafficking
AKAP9
OFD1
CDK5RAP2
Mitotic centrosomes
CYP3A5
CYP2C19
CYP2B6
Xenbiotic metabolism
mRNA Splicing
Cytoskeleton
Vesicle trafficking
Mitotic centrosomes
Xenbiotic metabolism
E
Gene overlap by Whippet
Early vs Late
Normal vs Early
Normal vs Late
F
Network of enriched terms by cluster ID
Network of terms by differential AS
covalent chromatin modification
Metabolism of lipids
monocarboxylic acid metabolic process
carbohydrate derivative biosynthetic process
regulation of peptidase activity
small molecule catabolic process
Complement and coagulation cascades
cellular response to organonitrogen compound
Vesicle-mediated transport
response to endoplasmic reticulum stress
organic hydroxy compound metabolic process
regulation of body fluid levels
protein targeting
Phospholipase D signaling pathway
regulation of mitotic cell cycle
leukocyte differentiation
The role of Nef in HIV-1 replication and disease patho
Sphingolipid signaling pathway
cell activation involved in immune response
response to leukemia inhibitory factor
Normal vs Early
Normal vs Late
Early vs Late
G
Pathway enrichment
-log10(P)
hsa04071: Sphingolipid signaling pathway
GO:0034976: response to endoplasmic reticulum stress
GO:0002521: leukocyte differentiation
GO:0052547: regulation of peptidase activity
GO:0007346: regulation of mitotic cell cycle
GO:0006605: protein targeting
GO:0016569: covalent chromatin modification
hsa04610: Complement and coagulation cascades
R-HSA-164952: The role of Nef in HIV-1 replication and disease pathogenesis
GO:0071417: cellular response to organonitrogen compound
R-HSA-5653656: Vesicle-mediated transport
GO:1990823: response to leukemia inhibitory factor
GO:0050878: regulation of body fluid levels
GO:0032787: monocarboxylic acid metabolic process
GO:0002263: cell activation involved in immune response
GO:1901137: carbohydrate derivative biosynthetic process
R-HSA-556833: Metabolism of lipids
GO:0044282: small molecule catabolic process
GO:1901615: organic hydroxy compound metabolic process
hsa04072: Phospholipase D signaling pathway
Normal vs Early
Normal vs Late
Early vs Late
H
Protein interaction network for genes with entropy >2
EIF4G1
RBM39
TMPO
MAPK9
CHD4
CTTN
EXOSC10
CTNNB1
CFL1
GORASP1
NUMA1
CSNK1D
HAUS4
HAUS2
CNTRL
ERCC3
RPS7
RPS6KB2
POLD4
HMGN1
RPL17
RFC4
RPL32
SFPQ
YWHAZ
FGFR2
HGF
FGFR3
FGFR1
PTBP1
SRSF5
U2AF1L4
POLR2D
SRSF7
FUS
POLR2H
NCBP2
HNRNPA2B1
PRPF3
HNRNPH1
PCBP2
RNPS1
TRAPPC3
TRAPPC6B
GOSR2
TRAPPC4
FLNA
CD44
FLOT1
GALT
ASL
ME2
PXN
RPLP0
FN1
RPS16
RPS24
RPL13
CUL1
EED
DNM1L
DIABLO
ATXN3
MKNK1
MALT1
CREBBP
ERC1
DYRK1A
APP
ZXDC
NR1H3
CREB1
SREBF2
SREBF1
M-phase
PI3K-Akt signaling
mRNA splicing
Ribosome and translation
Proteolysis and apoptosis
Stress response
ER Golgi trafficking
cell adhesion
lipid biosynthesis

Supplemental Figure 2: Metascape analysis of alternative splicing analyzed by MAJIQ or Whippet. A: Circos plot showing overlap of genes harboring the altered SEs in pairwise comparisons of the three clusters using MAJIQ. Purple lines connect the same genes. B: Network of enriched GO terms derived from genes harboring altered SEs according to MAJIQ. Left panel shows individual network clusters colored by functional annotation. Right panel shows networks colored by differential alternative splicing in pair-wise comparisons. C: Pathway enrichment for each pairwise comparison. D: Protein interaction networks colored by MCODE functional annotation (left) or differential alternative splicing (right). E: Circos plot showing overlap of genes harboring the altered SEs in pairwise comparisons of the three clusters using Whippet. F: Network of enriched GO terms derived from genes harboring altered SEs according to Whippet. Left panel shows individual network clusters colored by functional annotation. Right panel shows networks colored by differential alternative splicing in pair-wise comparisons. G: Pathway enrichment for each pairwise comparison by Whippet. H: Protein interaction networks for Genes with altered SEs with entropy >2 colored by MCODE functional annotation.

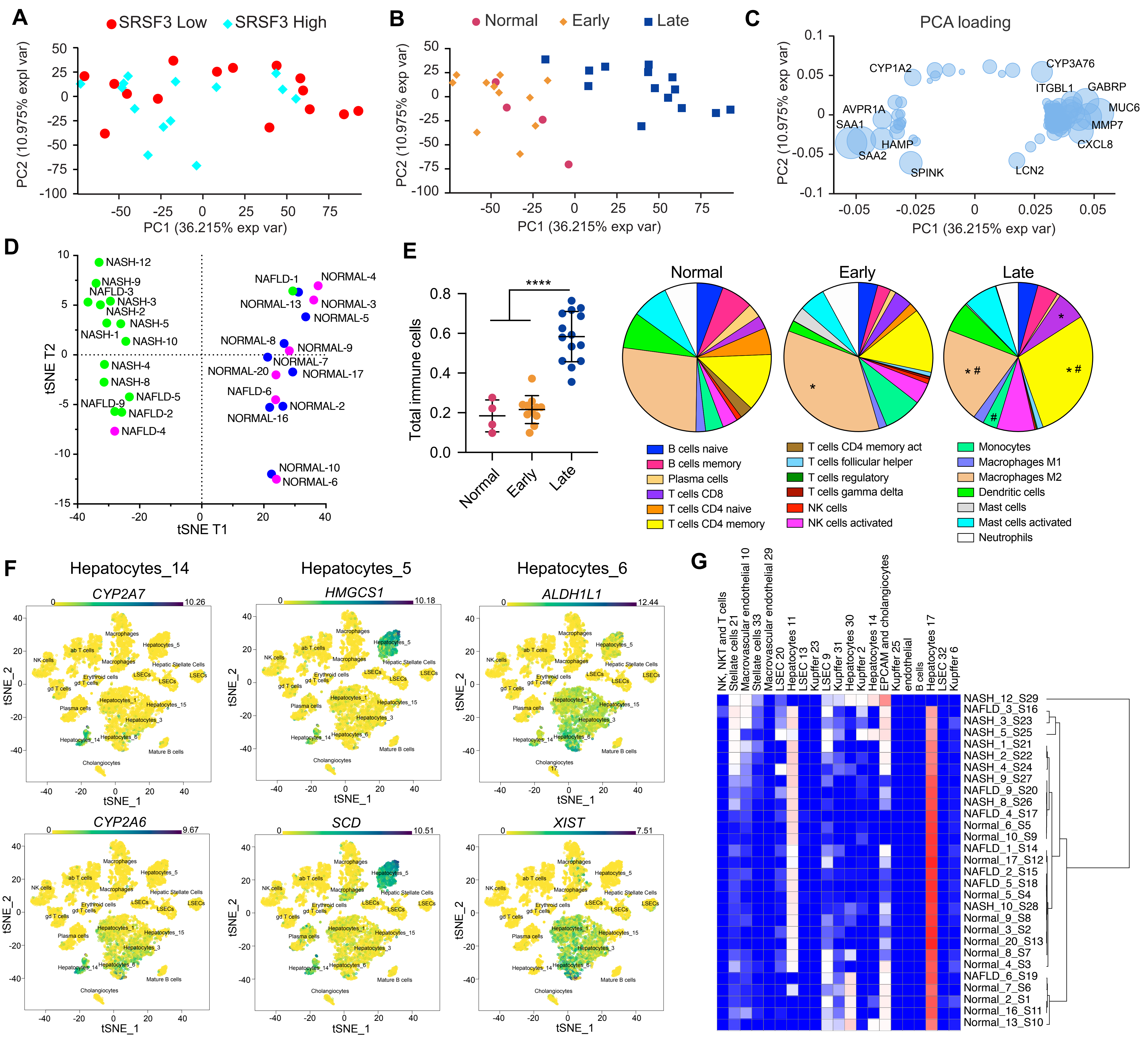

A
SRSF3 Low
SRSF3 High
PC2 (10.975% expl var)
PC1 (36.215% exp var)
B
Normal
Early
Late
PC2 (10.975% exp var)
PC1 (36.215% exp var)
C
PCA loading
PC2 (10.975% exp var)
PC1 (36.215% exp var)
CYP1A2
CYP3A76
ITGBL1
GABRP
MUC6
MMP7
CXCL8
AVPR1A
SAA1
HAMP
SAA2
SPINK
LCN2
D
tSNE T2
tSNE T1
E
Total immune cells
Normal
Early
Late
B cells naive
B cells memory
Plasma cells
T cells CD8
T cells CD4 naive
T cells CD4 memory
T cells CD4 memory act
T cells follicular helper
T cells regulatory
T cells gamma delta
NK cells
NK cells activated
Monocytes
Macrophages M1
Macrophages M2
Dendritic cells
Mast cells
Mast cells activated
Neutrophils
F
Hepatocytes_14
Hepatocytes_5
Hepatocytes_6
CYP2A7
HMGCS1
ALDH1L1
CYP2A6
SCD
XIST
tSNE_2
tSNE_1
G

Supplemental Figure 3: Transcriptome analysis of NAFLD and NASH samples. A: PCA plot based on gene expression across all 29 samples colored by SRSF3 expression. B: PCA plot colored by splicing clusters. C: Plot showing top 100 genes that contribute the most to the PCA. Each gene is shown by a blue circle, the size of the circle denoting the magnitude of the contribution. Top thirteen genes are labeled. D: Analysis of gene expression by tSNE. Samples are colored according to original designation: Normal = no history of liver disease. E: Graph shows total immune cell content after deconvolution of the data using CIBERSORT. Pie charts show immune cells fractions for individual splicing clusters. Asterisks indicated statistical significance vs Cluster A, # indicates significance vs Cluster B by t-test with *,# $p<0.05$. F: Single cell tSNE maps derived using the HumanLiver package in R developed by the Bader lab [44]. Maps are colored according to expression of CYP2A7 and CYP2A6 genes enriched in Hepatocyte cluster 14, HMGCS1 and SCD genes enriched in Hepatocyte cluster 5, and ALDH1L1 and XIST genes enriched in Hepatocyte cluster 6. Yellow signifies low expression, dark blue high expression. G: Heatmap showing liver cell composition based on cell signatures derived from the Human Liver Cell Atlas (http://human-liver-cell-atlas.ie-freiburg.mpg.de/). Blue indicates low relative cell content, red high.

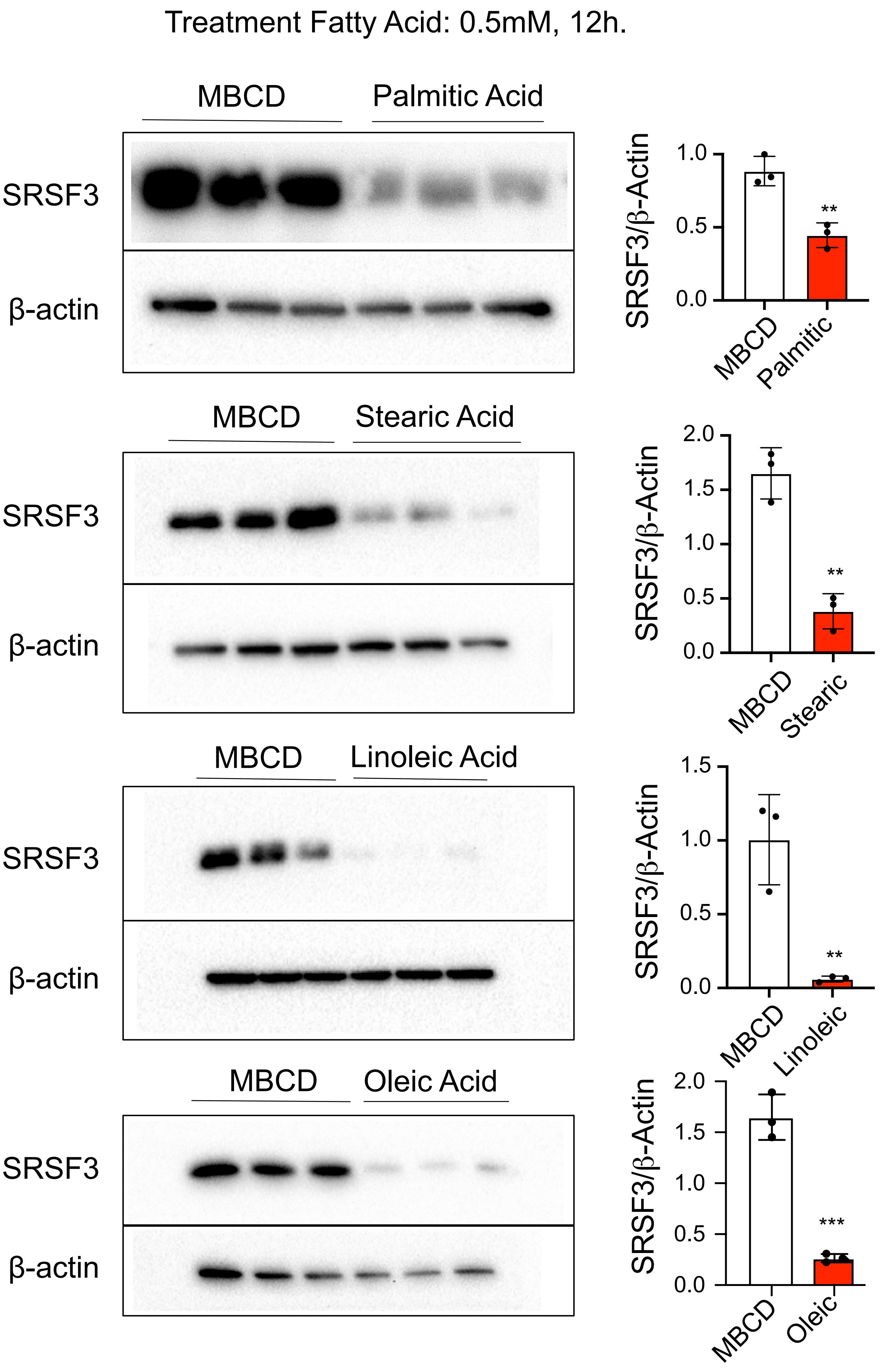
Treatment Fatty Acid: 0.5mM, 12h.
MBCD
Palmitic Acid
SRSF3
β-actin
SRSF3/β-Actin
MBCD
Palmitic
**
MBCD
Stearic Acid
SRSF3
β-actin
SRSF3/β-Actin
MBCD
Stearic
**
MBCD
Linoleic Acid
SRSF3
β-actin
SRSF3/β-Actin
MBCD
Linoleic
**
MBCD
Oleic Acid
SRSF3
β-actin
SRSF3/β-Actin
MBCD
Oleic
***

Supplemental Figure 4: Fatty acid treatment reduces SRSF3 protein levels in HepG2 cells. Hep G2 cells were treated with 500 μM of the four major fatty acids in the high fat diet, palmitic acid, stearic acid, linoleic acid or oleic acid, for 12 h. Fatty acid were solubilized using methyl-β-cyclodextrin (MBCD) which was used as a vehicle control. SRSF3 levels were measured by western blot and normalized to β-actin.

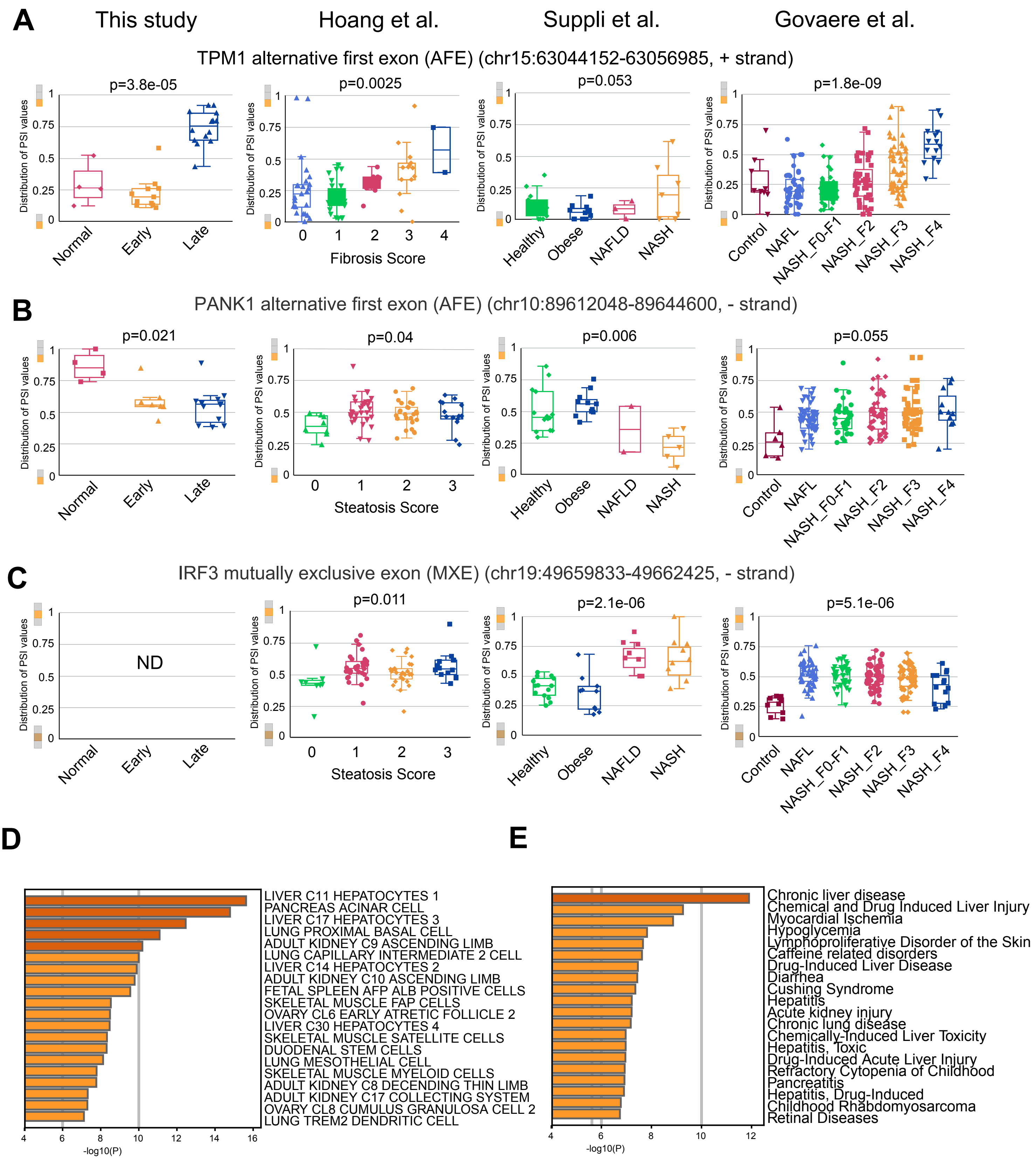
A
This study
Hoang et al.
Suppli et al.
Govaere et al.
TPM1 alternative first exon (AFE) (chr15:63044152-63056985, + strand)
p=3.8e-05
p=0.0025
p=0.053
p=1.8e-09
Distribution of PSI values
Normal
Early
Late
Fibrosis Score
Healthy
Obese
NAFLD
NASH
Control
NAFL
NASH_F0-F1
NASH_F2
NASH_F3
NASH_F4
B
PANK1 alternative first exon (AFE) (chr10:89612048-89644600, - strand)
p=0.021
p=0.04
p=0.006
p=0.055
Steatosis Score
C
IRF3 mutually exclusive exon (MXE) (chr19:49659833-49662425, - strand)
ND
p=0.011
p=2.1e-06
p=5.1e-06
D
LIVER C11 HEPATOCYTES 1
PANCREAS ACINAR CELL
LIVER C17 HEPATOCYTES 3
LUNG PROXIMAL BASAL CELL
ADULT KIDNEY C9 ASCENDING LIMB
LUNG CAPILLARY INTERMEDIATE 2 CELL
LIVER C14 HEPATOCYTES 2
ADULT KIDNEY C10 ASCENDING LIMB
FETAL SPLEEN AFP ALB POSITIVE CELLS
SKELETAL MUSCLE FAP CELLS
OVARY CL6 EARLY ATRETIC FOLLICLE 2
LIVER C30 HEPATOCYTES 4
SKELETAL MUSCLE SATELLITE CELLS
DUODENAL STEM CELLS
LUNG MESOTHELIAL CELL
SKELETAL MUSCLE MYELOID CELLS
ADULT KIDNEY C8 DECENDING THIN LIMB
ADULT KIDNEY C17 COLLECTING SYSTEM
OVARY CL8 CUMULUS GRANULOSA CELL 2
LUNG TREM2 DENDRITIC CELL
-log10(P)
E
Chronic liver disease
Chemical and Drug Induced Liver Injury
Myocardial Ischemia
Hypoglycemia
Lymphoproliferative Disorder of the Skin
Caffeine related disorders
Drug-Induced Liver Disease
Diarrhea
Cushing Syndrome
Hepatitis
Acute kidney injury
Chronic lung disease
Chemically-Induced Liver Toxicity
Hepatitis, Toxic
Drug-Induced Acute Liver Injury
Refractory Cytopenia of Childhood
Pancreatitis
Hepatitis, Drug-Induced
Childhood Rhabdomyosarcoma
Retinal Diseases
-log10(P)

Supplemental figure 5: Comparison of selected splicing events across four datasets. A: Splicing of TPM1alternative first exon across the four datasets (this study, Hoang et al, Suppli et al. or Govaere et al.). Kruskal-Wallis p-value is given for each dataset. Hoang et al. data is shown based on fibrosis score. B: Splicing of PANK1 alternative first exon across the four datasets Hoang et al. data is shown based on steatosis score. C: Splicing of IRF3 mutually exclusive exon across the four datasets Hoang et al. data is shown based on steatosis score. D: Analysis of the cell of origin for the genes harboring the 136 SEs that are common to the four datasets. E: Analysis of the disease associations for the genes harboring the 136 SEs that are common to the four datasets.

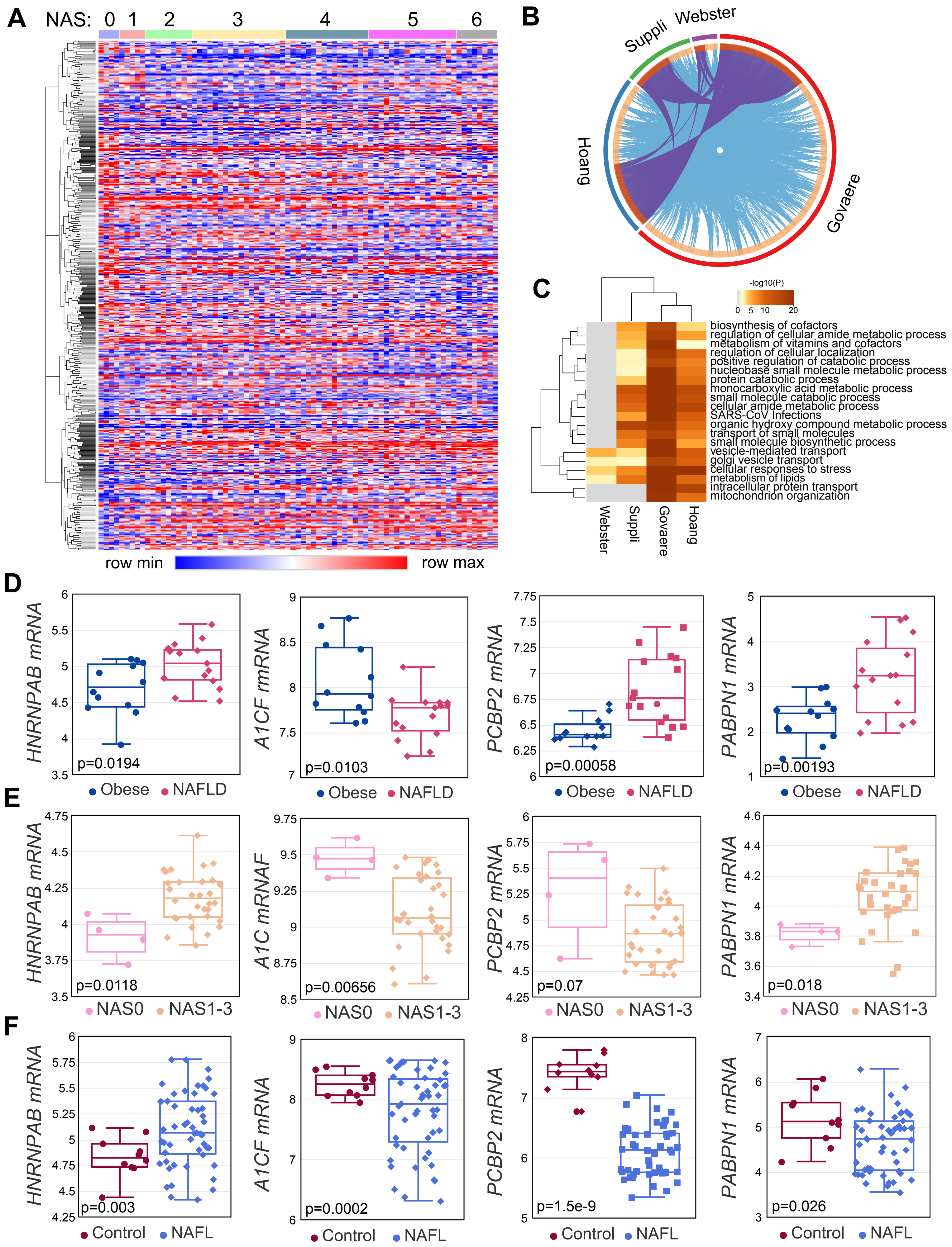

A
NAS: 0 1 2 3 4 5 6
row min
row max
B
Suppli
Webster
Hoang
Govaere
C
-log10(P)
0 5 10 20
biosynthesis of cofactors
regulation of cellular amide metabolic process
metabolism of vitamins and cofactors
regulation of cellular localization
positive regulation of catabolic process
nucleobase small molecule metabolic process
protein catabolic process
monocarboxylic acid metabolic process
small molecule catabolic process
cellular amide metabolic process
SARS-CoV Infections
organic hydroxy compound metabolic process
transport of small molecules
small molecule biosynthetic process
vesicle-mediated transport
golgi vesicle transport
cellular responses to stress
metabolism of lipids
intracellular protein transport
mitochondrion organization
Webster
Suppli
Govaere
Hoang
D
HNRNPAB mRNA
p=0.0194
Obese NAFLD
A1CF rmRNA
p=0.0103
Obese NAFLD
PCBP2 mRNA
p=0.00058
Obese NAFLD
PABPN1 mRNA
p=0.00193
Obese NAFLD
E
HNRNPAB mRNA
p=0.0118
NAS0 NAS1-3
A1C mRNAF
p=0.00656
NAS0 NAS1-3
PCBP2 mRNA
p=0.07
NAS0 NAS1-3
PABPN1 mRNA
p=0.018
NAS0 NAS1-3
F
HNRNPAB mRNA
p=0.003
Control NAFL
A1CF mRNA
p=0.0002
Control NAFL
PCBP2 mRNA
p=1.5e-9
Control NAFL
PABPN1 mRNA
p=0.026
Control NAFL

Supplemental Figure 6: Early changes in splicing. A: Heatmap for the top 500 SEs in Hoang et al., after filtering to remove SEs found in less than half the healthy control samples, or with PSI variances less than 0.001 (0.1% of max PSI). Samples are clustered according to the NAFLD activity score (NAS). Blue indicates low PSI, red high. B: Circos plot showing the overlap of genes harboring the altered SEs in the four studies. Purple lines indicate shared genes, blue lines indicate shared pathways or functions. C: Enrichment of GO terms for genes harboring the altered SEs from the four datasets. Color indicates significance (-log10P). D: Expression of the common early splicing factors *HNRNPAB, A1CF, PCBP2* and *PABPN1* in obese and NAFLD groups in Suppli et al. E: Expression of *HNRNPAB, A1CF, PCBP2* and *PABPN1* in NAS0 vs NAS1-3 groups in Hoang et al.. F: Expression of *HNRNPAB, A1CF, PCBP2* and *PABPN1* in control and NAFLD groups in Govaere et al.

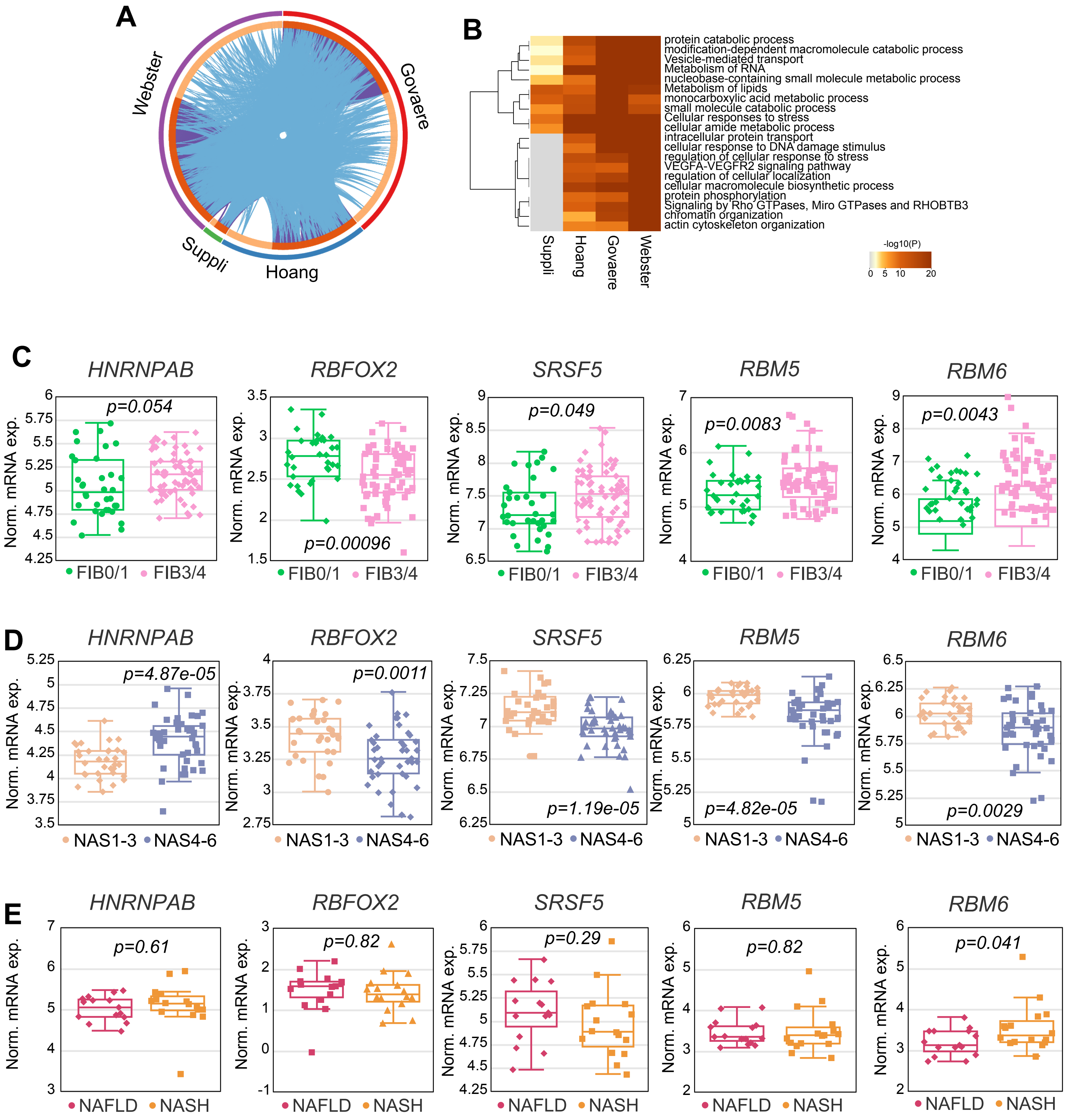
A
Webster
Govaere
Suppli
Hoang
B
protein catabolic process
modification-dependent macromolecule catabolic process
Vesicle-mediated transport
Metabolism of RNA
nucleobase-containing small molecule metabolic process
Metabolism of lipids
monocarboxylic acid metabolic process
small molecule catabolic process
Cellular responses to stress
cellular amide metabolic process
intracellular protein transport
cellular response to DNA damage stimulus
regulation of cellular response to stress
VEGFA-VEGFR2 signaling pathway
regulation of cellular localization
cellular macromolecule biosynthetic process
protein phosphorylation
Signaling by Rho GTPases, Miro GTPases and RHOBTB3
chromatin organization
actin cytoskeleton organization
Suppli
Hoang
Govaere
Webster
-log10(P)
0 5 10 20
C
HNRNPAB
p=0.054
RBFOX2
p=0.00096
SRSF5
p=0.049
RBM5
p=0.0083
RBM6
p=0.0043
Norm. mRNA exp.
FIB0/1
FIB3/4
D
HNRNPAB
p=4.87e-05
RBFOX2
p=0.0011
SRSF5
p=1.19e-05
RBM5
p=4.82e-05
RBM6
p=0.0029
Norm. mRNA exp.
NAS1-3
NAS4-6
E
HNRNPAB
p=0.61
RBFOX2
p=0.82
SRSF5
p=0.29
RBM5
p=0.82
RBM6
p=0.041
Norm. mRNA exp.
NAFLD
NASH

Supplemental Figure 7: Late changes in splicing. A: Circos plot showing the overlap of genes harboring the altered late SEs in the four studies. Purple lines indicate shared genes, blue lines indicate shared pathways or functions. C: Enrichment of GO terms for genes harboring the altered late SEs from the four datasets. Color indicates significance (-log10P). D: Expression of *HNRNPAB, RBFOX2, SRSF5, RBM5* and *RBM6* in FIB0/1 vs FIB3/4 groups in Hoang et al.. E: Expression of *HNRNPAB, RBFOX2, SRSF5, RBM5* and *RBM6* in NAS1-3 and NAS4-6 groups in Govaere et al. F: Expression of the common late splicing factors *HNRNPAB, RBFOX2, SRSF5, RBM5* and *RBM6* in NAFLD and NASH groups in Suppli et al.

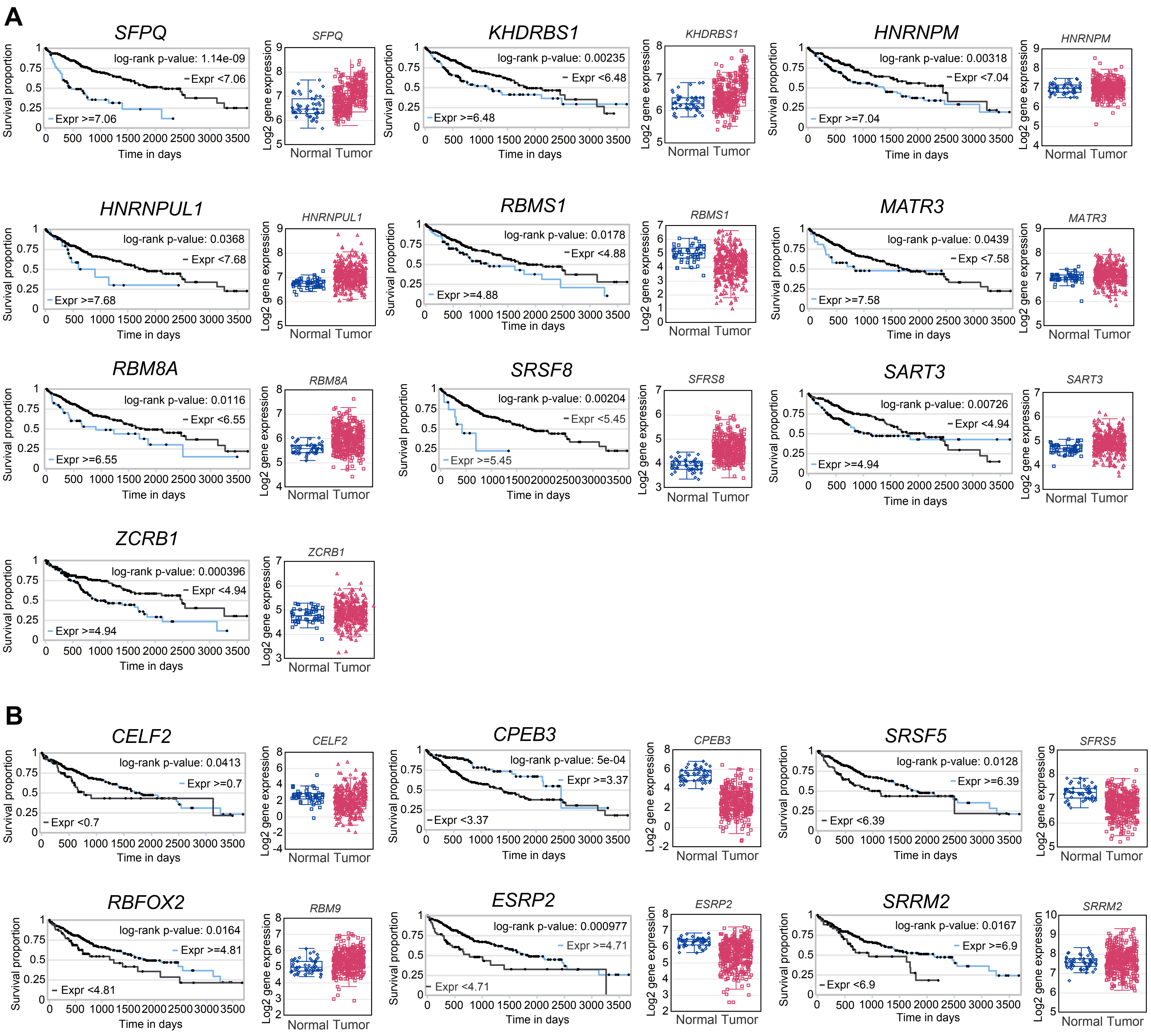
A
SFPQ
log-rank p-value: 1.14e-09
Expr <7.06
Expr >=7.06
KHDRBS1
log-rank p-value: 0.00235
Expr <6.48
Expr >=6.48
HNRNPM
log-rank p-value: 0.00318
Expr <7.04
Expr >=7.04
HNRNPUL1
log-rank p-value: 0.0368
Expr <7.68
Expr >=7.68
RBMS1
log-rank p-value: 0.0178
Expr <4.88
Expr >=4.88
MATR3
log-rank p-value: 0.0439
Exp <7.58
Expr >=7.58
RBM8A
log-rank p-value: 0.0116
Expr <6.55
Expr >=6.55
SRSF8
log-rank p-value: 0.00204
Expr <5.45
Expr >=5.45
SFRS8
SART3
log-rank p-value: 0.00726
Expr <4.94
Expr >=4.94
ZCRB1
log-rank p-value: 0.000396
Expr <4.94
Expr >=4.94
B
CELF2
log-rank p-value: 0.0413
Expr >=0.7
Expr <0.7
CPEB3
log-rank p-value: 5e-04
Expr >=3.37
Expr <3.37
SRSF5
log-rank p-value: 0.0128
Expr >=6.39
Expr <6.39
SFRS5
RBFOX2
log-rank p-value: 0.0164
Expr >=4.81
Expr <4.81
RBM9
ESRP2
log-rank p-value: 0.000977
Expr >=4.71
Expr <4.71
SRRM2
log-rank p-value: 0.0167
Expr >=6.9
Expr <6.9
Survival proportion
Time in days
Log2 gene expression
Normal Tumor

Supplemental Figure 8: Survival curves for common splicing factors. A: Kaplan-Meier survival curves for HCC patients and gene expression of splicing factors *SFPQ, KHDRBS1, HNRNPM, HNRNPUL1, RBMS1, MATR3, RBM8A, SRSF8, SART3* and *ZCRB1* where lower expression is associated with better survival. B: Kaplan-Meier survival curves for HCC patients and gene expression of splicing factors *CELF2, CPEB3, SRSF5, RBFOX*2*, ESRP2, and SRRM2* where higher expression is associated with better survival. Log-rank p-values are given.

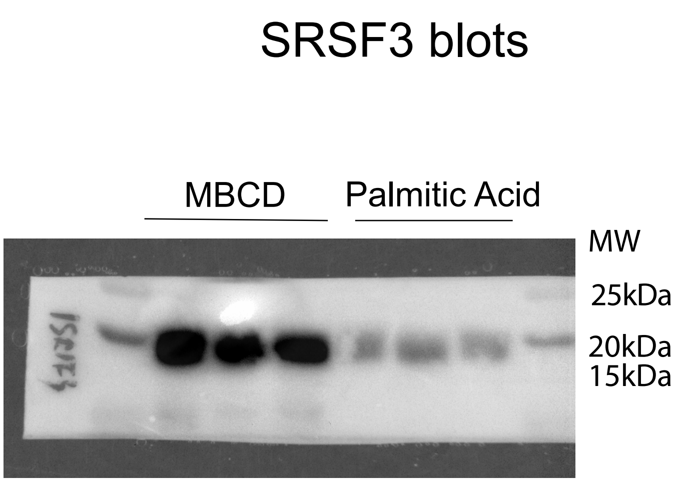
SRSF3 blots
MBCD
Palmitic Acid
MW
25kDa
20kDa
15kDa

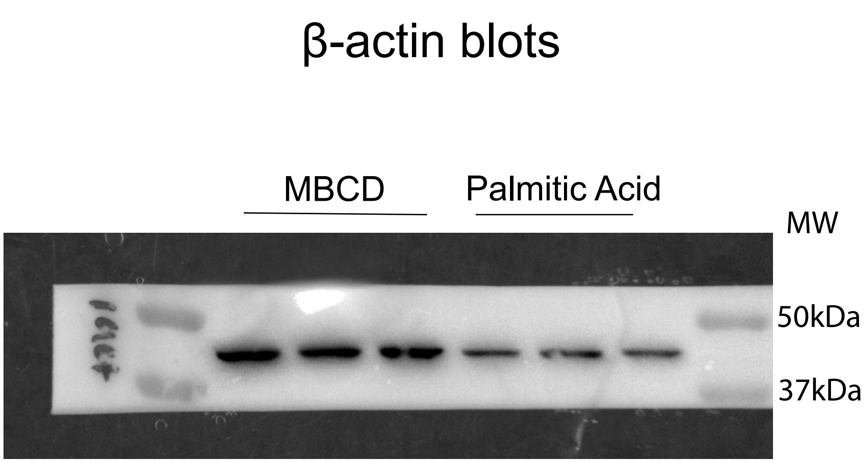
β-actin blots
MBCD
Palmitic Acid
MW
50kDa
37kDa

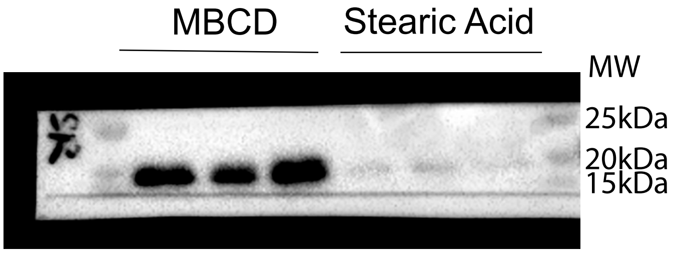
MBCD
Stearic Acid
MW
25kDa
20kDa
15kDa

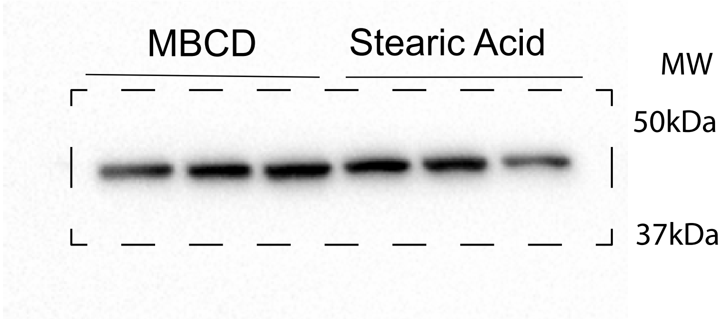
MBCD
Stearic Acid
MW
50kDa
37kDa

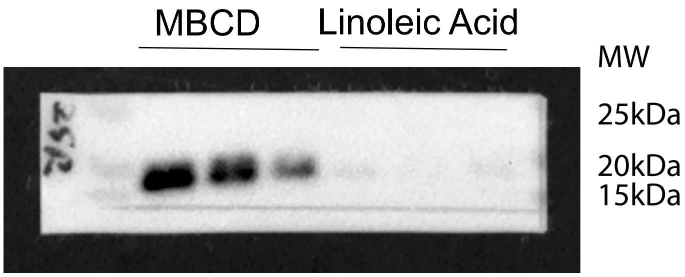
MBCD
Linoleic Acid
MW
25kDa
20kDa
15kDa

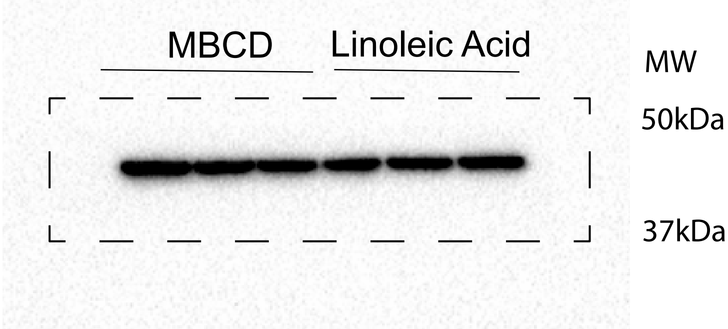
MBCD
Linoleic Acid
MW
50kDa
37kDa

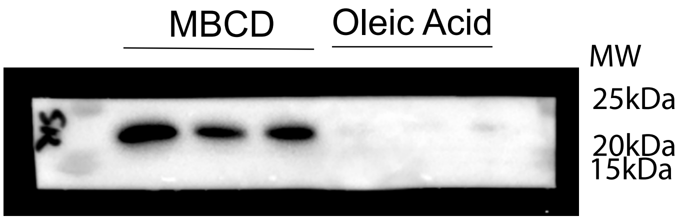
MBCD
Oleic Acid
MW
25kDa
20kDa
15kDa

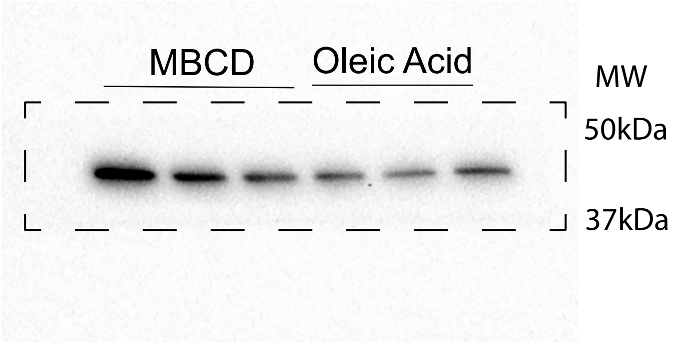
MBCD
Oleic Acid
MW
50kDa
37kDa

Supplemental Figure 9: Original blots for Figure S4. Equal amounts of protein were separated by SDS-PAGE and transferred to PVDF membranes. Membranes are cut below the 37kDa marker and above the 50 kDa marker (pre-stained markers) so that SRSF3 and β-actin could be quantified from the same gel. The lower portion was used for immunoblotting for SRSF2 (21 kDa) and the upper portion used for immunoblotting for β-actin (42 kDa) as a loading control. Chemiluminescence and brightfield images were taken of the SRSF3 blots (left panels) and merged to allow positioning of the molecular weight markers relative to the SRSF3 protein. β-actin always runs between the 37 and 50 kDa MW markers so merged images are not routinely taken, only chemiluminescence. Top panel for palmitic acid shows a representative merged images for β-actin (right panels). Dotted outlines below show the dimensions of the membranes in the chemiluminescence images.
